# Non-PML neuroinvasive infections reported with multiple sclerosis disease-modifying therapies: a mechanism-based pharmacovigilance study, 2004–2025

**DOI:** 10.3389/fimmu.2026.1959164

**Published:** 2026-10-13

**Authors:** Li Ke, Sili Jiang, Jian Duan, Wenli Xing

**Affiliations:** Department of Cerebrovascular Diseases, Suining Central Hospital, Suining, Sichuan, China

**Keywords:** disease-modifying therapy, disproportionality analysis, FAERS (FDA Adverse Event Reporting System), multiple sclerosis, neuroinvasive infection, pharmacovigilance

## Abstract

**Background:**

Multiple sclerosis (MS) disease-modifying therapies (DMTs) alter host immunity and may produce pathogen-specific susceptibility to neuroinvasive infection. Comparative evidence is limited because events are rare and postmarketing research focuses mainly on progressive multifocal leukoencephalopathy (PML).

**Methods:**

We analyzed deduplicated US FDA Adverse Event Reporting System (FAERS) reports from 2004Q1 to 2025Q4 using a prespecified case–non-case design. Tier A comprised direct high-certainty non-PML neuroinvasive infection terms. Tier B comprised a possible CNS infectious syndrome plus pathogen evidence elsewhere in the report, without CNS confirmation; tier A+B was a lower-certainty expanded endpoint for sensitivity and pathogen composition analyses. We estimated overall reporting (E1), pathogen composition within tier A+B (E2), and same-pathogen CNS localization (E3). Five DMT mechanisms were compared with contemporaneous active DMTs using calendar-year Mantel–Haenszel reporting odds ratios and covariate-adjusted Jeffreys-prior penalized logistic models.

**Results:**

Among 492,514 reports, 675 were tier A and 82 additional reports were tier B. Tier A aRORs (95% CIs) were 0.82 (0.63–1.05) for anti-CD20 therapies, 1.77 (1.44–2.16) for S1P modulators, 1.32 (1.06–1.64) for natalizumab, 3.28 (2.24–4.66) for alemtuzumab, and 0.56 (0.21–1.19) for cladribine. Fingolimod accounted for 145 of 152 S1P cases and had an aROR of 2.15 (1.75–2.64). Among four mechanisms eligible for E2, pathogen composition differed (LRT 210.59, 9 *df*; bootstrap *P* = 9.99 × 10^-4^): viral reports were enriched with anti-CD20 therapies (OR 4.24, 2.56–7.02) and natalizumab (2.03, 1.26–3.26), fungal reports with S1P modulators (29.09, 15.77–53.64), and bacterial reports with alemtuzumab (4.99, 2.27–11.01). Only natalizumab–HSV/VZV met the adjusted E3 gate and showed CNS enrichment (aROR 2.80, 1.80–4.38; Holm-adjusted *P* = 5.81 × 10^-6^).

**Conclusions:**

E1 contrasts were interpreted as separate reporting comparisons, not a formal equality test across mechanisms. S1P reporting was largely fingolimod-driven, and natalizumab–HSV/VZV was the only prespecified pair with adjusted same-pathogen CNS enrichment. Findings support targeted vigilance and confirmatory studies but do not estimate incidence or establish causality.

## 1 Introduction

Multiple sclerosis (MS) is a chronic immune-mediated disease of the central nervous system (CNS) and a major cause of non-traumatic neurological disability in young adults. The third Atlas of MS estimated that 2.8 million people were living with MS worldwide, with prevalence increasing in every region (1). Its pathobiology spans peripheral activation and trafficking of adaptive immune cells and compartmentalized CNS inflammation involving B cells, T cells, myeloid cells, astrocytes, and microglia (2). Therapies directed at these pathways may therefore disrupt host defense in different ways (3).

Disease-modifying therapies (DMTs) can strongly suppress inflammatory disease activity, and high-efficacy agents are increasingly used earlier in selected patients (4, 5). Comparative evidence suggests that early intensive treatment may reduce disability worsening relative to escalation, although long-term safety comparisons remain uncertain (6). Network meta-analyses also identify clinically relevant differences in efficacy and tolerability across agents (7). Safety rankings among high-efficacy therapies depend on the outcome definition and duration of follow-up (8). Treatment selection must therefore balance suppression of MS activity against uncommon, delayed, and potentially pathogen-specific adverse effects, recognizing that the consequences of uncontrolled disease activity may be irreversible.

Infection is among the most consequential complications of DMT exposure. An ECTRIMS-focused workshop concluded that risk varies with the immune target, treatment duration, host factors, previous therapy, and pathogen (9). Consensus recommendations accordingly emphasize treatment-specific screening, vaccination, prevention, and surveillance (10, 11). Clinical and population-based studies associate selected DMTs with infection-related outcomes, but estimates vary across trials, healthcare databases, and treatment contrasts (12). A recent systematic review found few observational comparisons and considerable heterogeneity, including for neurological infections (13).

The pharmacology of individual DMTs provides a basis for distinct neuroinvasive infection profiles. Anti-CD20 antibodies produce sustained B-cell depletion and can compromise humoral immunity through treatment-emergent hypogammaglobulinemia (14). Sphingosine-1-phosphate (S1P) receptor modulators sequester lymphocytes by impairing their egress from lymphoid tissue, with product-specific differences in receptor selectivity and pharmacokinetics (15). Natalizumab blocks α4-integrin-dependent leukocyte trafficking across the blood–brain barrier and thereby alters CNS immune surveillance (16). Alemtuzumab produces profound CD52-positive lymphocyte depletion followed by asynchronous immune reconstitution (17). Cladribine is a pulsed immune-reconstitution therapy in which lymphopenia and herpes zoster are reported more consistently than broad opportunistic infection (18). These mechanistic differences may influence the occurrence, pathogen class, and anatomical distribution of reported infections.

Postmarketing research on neuroinfection in MS has been dominated by JC-virus-associated PML. Most published evidence concerns natalizumab, although PML has also been reported with other immune therapies (19). Far less is known about non-PML meningitis, encephalitis, meningoencephalitis, abscess, myelitis, vasculitis, and related syndromes. Spontaneous-reporting systems can reveal rare or unexpected patterns, but valid interpretation requires transparent outcomes, appropriate comparators, calendar-time control, and a clear distinction between disproportionality, incidence, and causality (20). We therefore examined five major MS-DMT mechanisms in the US Food and Drug Administration Adverse Event Reporting System (FAERS). The prespecified analysis sought to characterize mechanism-specific tier A reporting estimates (E1), test whether pathogen composition varied by mechanism (E2), and assess whether selected pathogens were preferentially localized to the CNS (E3). E1 comprised separate target-versus-pooled-reference contrasts and was not a formal cross-mechanism equality test.

## 2 Materials and methods

### 2.1 Study design and reporting framework

We conducted a retrospective pharmacovigilance case–non-case study using quarterly FAERS data from 2004Q1 to 2025Q4. Study design and reporting followed the READUS-PV statement and its explanation and elaboration (21, 22). Before analysis, we locked the protocol, exposure and outcome dictionaries, estimands, model gates, multiplicity families, and principal sensitivity analyses.

FAERS contains deidentified spontaneous reports but lacks a complete population denominator and verified exposure time. We therefore use the terms “report,” “reporting signal,” “reporting odds,” and “reporting composition” throughout. RORs are not interpreted as incidence-rate ratios, risks, or causal effects.

### 2.2 Data source, deduplication, and cohort construction

We harmonized quarterly ASCII files across legacy AERS and FAERS formats and removed deleted reports. Records sharing a CASEID were reduced to the version with the latest FDA receipt date (FDA_DT), consistent with FDA deduplication principles. The highest PRIMARYID resolved ties. The latest deduplicated CASEID was the analysis unit; historical versions were retained for audit but were not counted as independent observations.

Drug names were mapped to generic products using a prespecified dictionary of brand names, spelling variants, and legacy names. Indications were normalized to identify MS and its subtypes. Cohort inclusion required an eligible MS indication linked to a mapped DMT; prespecified non-MS and product-use contexts were excluded. In the strict primary analysis, the target DMT had to be coded as primary suspect (PS), with no other MS DMT coded as PS or secondary suspect (SS) in the same report. A PS+SS definition assessed role misclassification. Reports were aligned to each product’s US marketing period, and comparisons included only DMTs available during that period.

For each target mechanism, the locked reference comprised reports from the target’s launch quarter onward in all other primary-mechanism or immune-other DMT groups, excluding interferon-beta/glatiramer acetate and anti-CD20 extension-only products. These pooled references were used in separate target-versus-reference models and do not constitute direct pairwise tests between mechanisms. Interferon-beta/glatiramer acetate and all mapped MS-DMT sets were evaluated as secondary comparators. Mechanism groups were anti-CD20 core (ocrelizumab/ofatumumab), S1P modulators, natalizumab, alemtuzumab, and cladribine; rituximab and ublituximab were retained only in the extension sensitivity group.

### 2.3 Neuroinvasive infection outcomes

Adverse events were coded using MedDRA version 29.0 preferred terms (PTs). The locked screening dictionary contained 1,648 PTs: 85 pathogen-specific CNS terms (A1), 27 explicitly infectious CNS syndromes (A2), 10 ambiguous CNS terms (tier C), 34 non-infectious mimics, 7 PML/JCV terms, and 1,485 additional terms used to capture extracranial or pathogen evidence (1,464 pathogen-coded terms and 21 immune/neurovascular proxy terms) (**Supplementary Table S1**; **Supplementary Data S1A**). The broad infection-flag union contained 1,580 PTs before endpoint exclusions and 1,576 after removal of four pathogen-coded PML/JCV PTs; it was used only for sensitivity/context analyses and did not redefine tier A. Tier A comprised direct, high-certainty non-PML neuroinvasive infections. Tier B used a prespecified combination algorithm in which a possible neurological infectious syndrome required co-reported pathogen evidence elsewhere in the report, without confirmation that the CNS was infected. Tier A+B was used for expanded-outcome and pathogen composition analyses. Tier C contained ambiguous terms and was restricted to exploratory analyses. Events explicitly coded as drug-induced, immune-mediated, aseptic, or otherwise non-infectious were excluded. PML and JC virus terms were excluded from the target outcome but retained for audit and positive-control analyses.

The locked dictionary classified pathogens as viral, fungal, bacterial, protozoan/parasite, mixed, or other/unspecified. To stabilize the global multinomial model, mixed and other/unspecified categories were combined, and sparse age and MS-subtype levels were consolidated as prespecified. Four drug–pathogen hypotheses were evaluated as prespecified mechanistic anchors: anti-CD20–enterovirus, S1P–*Cryptococcus*, natalizumab–HSV/VZV, and alemtuzumab–*Listeria*. They did not define the complete pathogen universe.

### 2.4 Estimands

We defined three estimands to address separate clinical questions (**Figure 1C**).

**Figure 1:**
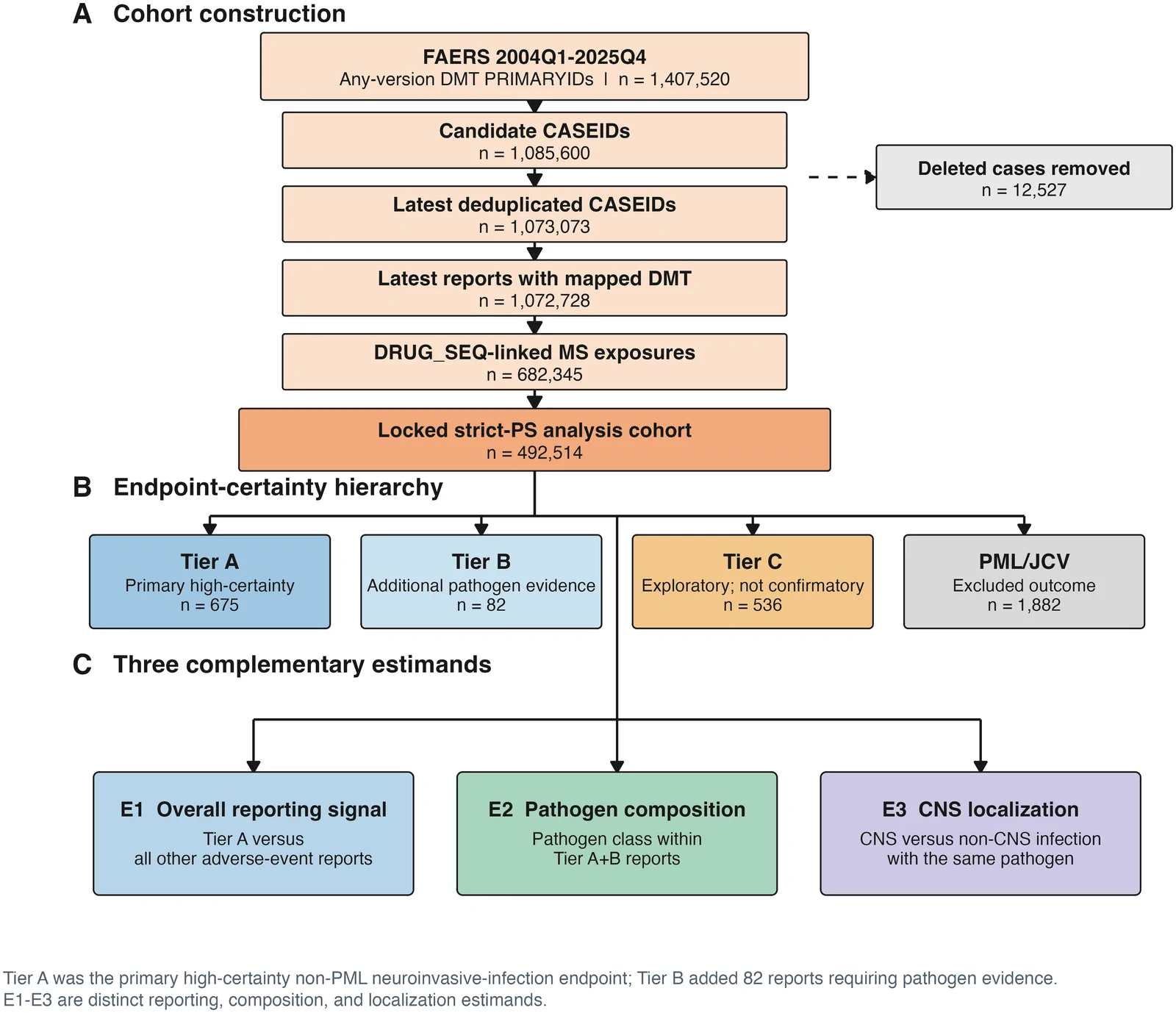
Study cohort, endpoint certainty, and analysis architecture. **(A)** Sequential cohort construction from all-version DMT PRIMARYIDs to the locked analysis cohort; the latest deduplicated CASEID was the analysis unit. **(B)** Endpoint-certainty hierarchy. Tier A comprised high-certainty non-PML neuroinvasive infections; tier B required a possible neurological infectious syndrome plus pathogen evidence elsewhere in the report and was lower certainty than tier A; tier C was exploratory and excluded from confirmatory analyses. PML/JC virus events were retained only for the excluded-outcome count and positive-control context. **(C)** Three complementary estimands: tier A versus all other adverse events (E1), pathogen composition within tier A+B reports (E2), and same-pathogen CNS versus non-CNS infection (E3).

1. E1, overall reporting disproportionality: tier A neuroinvasive infection versus all other adverse-event reports for a target mechanism compared with contemporaneous active MS-DMT reports.
2. E2, pathogen composition: the odds that a tier A+B infection report belonged to a specified pathogen class for a target mechanism relative to contemporaneous infection reports for comparator mechanisms. This is a case-only composition estimand and not an infection-incidence estimand.
3. E3, same-pathogen CNS localization: for each H1–H4 pathogen, CNS infection versus non-CNS infection with the same pathogen in the target mechanism compared with reference DMTs. This case–case estimand tests additional CNS enrichment conditional on pathogen reporting.

A *post hoc* analysis also compared each specific CNS drug–pathogen event with all other adverse-event reports. Because this estimand conditioned on neither infection nor the same pathogen, it was interpreted separately from E3.

### 2.5 Descriptive and disproportionality analyses

We summarized report counts, age, sex, region, MS subtype, reporter type, serious outcomes, and missingness by exposure group. Continuous variables are reported as medians and interquartile ranges (IQRs), and categorical variables as counts and proportions. Unknown age, sex, region, and subtype were retained as separate categories because missingness in spontaneous reports is unlikely to be random.

Crude RORs and 95% confidence intervals (CIs) were calculated from 2 × 2 tables. The ROR is a standard measure of relative reporting in spontaneous-reporting data (23). MH-RORs were stratified by calendar year to account for changes in product availability and reporting. The primary adjusted model used Jeffreys-prior penalized logistic regression with age group, sex, calendar year, region, and MS subtype as covariates. This approach yields finite, shrinkage-based estimates when data are sparse or separated (24). Main model 95% CIs were obtained by penalized profile likelihood; Wald intervals were used in specified sensitivity analyses. Models required at least 30 total outcome events and 10 reference events, and signal interpretation required at least 5 target events.

The five primary mechanism models formed one multiplicity family, with *P*-values adjusted by the Benjamini–Hochberg (BH) procedure. For E2, adjusted one-versus-rest logistic models were fitted within tier A+B reports for each mechanism–pathogen class that passed the prespecified size gate. These *P*-values were controlled with the BH false discovery rate procedure. An adjusted multinomial logistic model tested overall pathogen heterogeneity among mechanisms. The global likelihood-ratio statistic was calibrated with 1,000 parametric bootstrap replicates under the covariate-adjusted null model. An unadjusted Freeman–Halton Fisher test with 100,000 Monte Carlo replicates and Cramér’s *V* provided complementary global summaries.

For E3, crude and calendar-year MH-ROR *P*-values for H1–H4 were adjusted with Holm’s method. Covariate-adjusted Jeffreys-prior penalized models were fitted only when target and reference data met the prespecified same-pathogen gate of at least five CNS and five non-CNS reports. The four *post hoc* specific-event versus all-other-adverse-event models formed a separate multiplicity family.

### 2.6 Sensitivity, internal validity, and descriptive clinical analyses

Prespecified sensitivity analyses varied outcome certainty, exposure role, comparator set, reporter type, seriousness, geography, MS subtype, and age completeness. Additional analyses addressed co-reported corticosteroids or strong immunosuppressants, treatment carryover, the pandemic period, SARS-CoV-2 reporting, literature/study source, and suspected near-duplication. Outcome definitions were tier A+B and exploratory tier A+B+C; exposure definitions were strict PS, unique PS, and PS+SS. Leave-one-product-out analyses assessed whether a class signal depended on one product. Product-level S1P analyses and a common-period four-product heterogeneity test localized the class signal. Natalizumab–PML and S1P–herpes infection served as internal positive-control contexts. Definitions, construction audits, the complete sensitivity matrix, and the SARS-CoV-2 audit are provided in **Supplementary Tables S1–S4**.

Time to onset (TTO) was calculated only when complete, logically valid treatment-start and event dates were available; dates were not imputed. We summarized TTO using medians, IQRs, bootstrap CIs, and empirical cumulative distributions. Reports with and without usable dates were compared to assess selection into the TTO subset. Because FAERS has no censoring mechanism, Kaplan–Meier risks and hazards were not estimated. At the report level, serious_outcome was coded when any FAERS OUTC_COD was DE, LT, HO, DS, CA, RI, or OT; death was coded when DE was present. Multiple codes were collapsed to binary indicators. These descriptive fields are not adjudicated infection-attributable deaths or case fatality rates.

Analyses were conducted in R version 4.5.3 using data.**Table 1**.18.4, arrow 24.0.0, and brglm2 1.1.0. Statistical tests were two-sided. Exact adjusted *P*-values and confidence intervals are presented in the main and supplementary tables.

**Table 1:** Characteristics of the locked FAERS analysis cohort by MS-DMT exposure group.

Exposure group	Reports, *n*	Tier A, *n*	Tier A+B, *n*	Age, median (IQR), years	Age missing, %	Female, %	Sex missing, %	HCP reporter, %	United States, %	Relapsing MS, %	Reports with any serious outcome flag, *n*/*N* (%)	Reports with death flag, *n*/*N* (%)
Interferon-beta/glatiramer acetate	145,084	123	140	49 (39 to 58)	37.8	77.4	2.2	22.5	78.1	6.2	83,548/145,084 (57.6%)	7,649/145,084 (5.27%)
Other active/immune DMT	102,723	68	76	51 (42 to 59)	50.1	73.1	7.4	22.5	90.9	4.6	34,938/102,723 (34.0%)	1,974/102,723 (1.92%)
Anti-CD20 core	42,794	122	145	46 (37 to 56)	47.1	68.3	6.8	33.4	62.1	42.8	20,177/42,794 (47.1%)	917/42,794 (2.14%)
Anti-CD20 extension	3,203	11	12	43 (33 to 52)	59.9	38.7	45.7	62.5	62.6	17.3	1,469/3,203 (45.9%)	107/3,203 (3.34%)
S1P modulators	60,450	152	168	45 (36 to 54)	41.4	75.1	3	32.1	43.4	16.7	29,408/60,450 (48.6%)	814/60,450 (1.35%)
Natalizumab	132,099	159	171	46 (37 to 54)	34.6	75.9	1.2	15.3	92.2	2.4	35,250/132,099 (26.7%)	3,305/132,099 (2.50%)
Alemtuzumab	3,084	35	39	40 (32 to 48)	20.6	63.4	10.5	43.9	45.4	22	2,618/3,084 (84.9%)	91/3,084 (2.95%)
Cladribine	3,077	5	6	47 (37 to 57)	42.5	74.7	4	34.5	65.9	7.2	1,773/3,077 (57.6%)	82/3,077 (2.67%)

DMT, disease-modifying therapy; HCP, healthcare professional; IQR, interquartile range; MS, multiple sclerosis.

## 3 Results

### 3.1 Cohort, outcome certainty, and analysis architecture

The locked cohort comprised 492,514 unique deduplicated CASEIDs (**Figure 1A**; **Supplementary Table S2**). Exposure groups included 145,084 interferon-beta/glatiramer acetate reports and 102,723 other active/immune DMT reports. Target groups comprised 42,794 anti-CD20 core, 3,203 anti-CD20 extension, 60,450 S1P modulator, 132,099 natalizumab, 3,084 alemtuzumab, and 3,077 cladribine reports (**Table 1**). Median reported age ranged from 40 years for alemtuzumab to 51 years for other active/immune DMTs. Most exposure groups were predominantly female. Completeness of demographic data, reporter type, geographical distribution, and recorded MS subtype varied across mechanisms. Relapsing MS, for example, was recorded in 42.8% of anti-CD20 core reports and 2.4% of natalizumab reports.

We identified 675 tier A reports, 82 additional tier B reports, and 536 tier C reports. An additional 1,882 PML/JCV reports were excluded from the target outcome (**Figure 1B**). The primary tier A and expanded tier A+B analyses therefore included 675 and 757 reports, respectively. Serious outcome and death flags were report-level descriptive indicators: **Table 1** summarizes them among all reports in each exposure group, whereas **Supplementary Table S9** summarizes component outcomes among tier A+B reports. The three estimands, E1, E2, and E3, were analyzed separately as prespecified (**Figure 1C**).

**Table 1** outcome columns are report-level descriptive flags. Serious outcome denotes any FAERS OUTC_COD of DE, LT, HO, DS, CA, RI, or OT; death denotes DE. Percentages are calculated among all reports in each exposure group and are not infection-attributable case fatality, incidence, mortality, or causal estimates. The anti-CD20 extension row (rituximab/ublituximab) is a sensitivity group and is not part of the locked anti-CD20 core analysis.

### 3.2 Mechanism-specific tier A reporting estimates

Each model compared one target mechanism with its prespecified pooled contemporaneous reference; the five contrasts are separate target-versus-reference estimates and do not constitute a formal test of equality between mechanisms. Calendar-time and covariate adjustment changed several crude estimates (**Figure 2**; **Table 2**). Anti-CD20 core therapies had 122 tier A reports and 253 reference events, with no adjusted positive signal (aROR 0.82, 95% CI 0.63–1.05; BH-adjusted *P* = 0.144). S1P modulators had 152 target and 367 reference events and showed a positive signal (aROR 1.77, 1.44–2.16; BH-adjusted *P* = 5.32 × 10^-8^). For natalizumab, the crude ROR was below 1 (0.67, 0.55–0.80), but the direction changed after calendar-year stratification and covariate adjustment. The final aROR was 1.32 (1.06–1.64; BH-adjusted *P* = 0.020). Alemtuzumab had the largest adjusted estimate (35 target and 418 reference events; aROR 3.28, 2.24–4.66; BH-adjusted *P* = 5.60 × 10^-^¹^0^), although it contributed fewer target events than S1P modulators or natalizumab. Cladribine contributed five tier A reports and yielded an imprecise estimate (aROR 0.56, 0.21–1.19; BH-adjusted *P* = 0.177). The sparse cladribine data did not support a protective interpretation.

**Figure 2:**
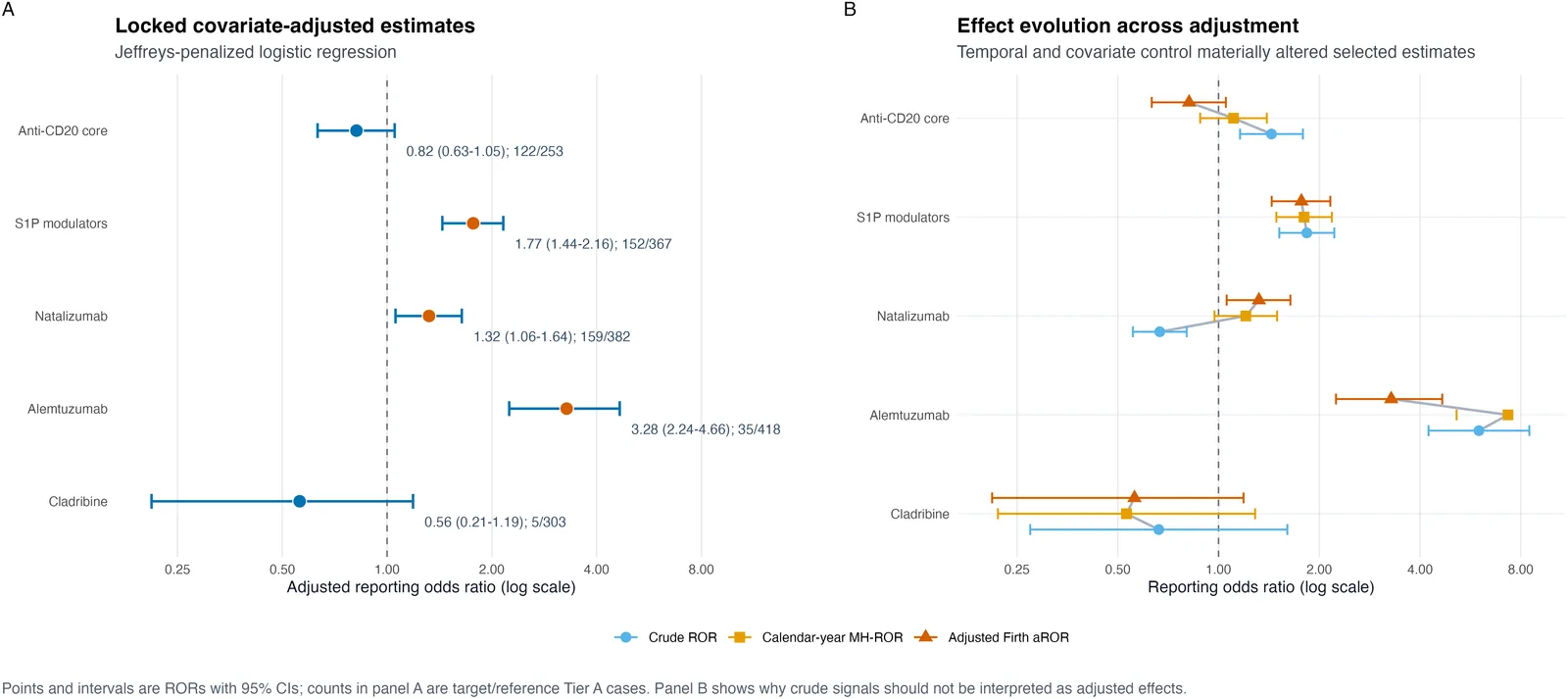
Primary tier A reporting disproportionality by DMT mechanism. **(A)** Locked aRORs and 95% CIs from Jeffreys-prior penalized logistic models comparing each target mechanism with contemporaneous active DMT comparators. Models adjusted for age group, sex, calendar year, region, and MS subtype; counts denote target/reference tier A cases. **(B)** Changes from crude ROR to calendar-year MH-ROR and covariate-adjusted aROR. Orange markers identify adjusted estimates with 95% CIs excluding 1; the dashed line marks the null.

**Table 2:** Primary tier A reporting disproportionality by DMT mechanism.

DMT mechanism	Target/reference tier A cases	Target/reference reports	Crude ROR (95% CI)	Calendar-year MH-ROR (95% CI)	Adjusted Firth aROR (95% CI)	Adjusted-model BH P	Evidence interpretation
Anti-CD20 core	122/253	42,794/127,615	1.44 (1.16 to 1.79)	1.11 (0.88 to 1.39)	0.82 (0.63 to 1.05)	0.144	No adjusted overall positive signal
S1P modulators	152/367	60,450/267,535	1.84 (1.52 to 2.22)	1.80 (1.49 to 2.18)	1.77 (1.44 to 2.16)	5.32 × 10^-8^	Positive; subsequently shown to be fingolimod-driven
Natalizumab	159/382	132,099/211,960	0.67 (0.55 to 0.80)	1.21 (0.97 to 1.50)	1.32 (1.06 to 1.64)	0.020	Moderate adjusted positive signal
Alemtuzumab	35/418	3,084/218,818	6.00 (4.24 to 8.48)	7.33 (5.14 to 10.43)	3.28 (2.24 to 4.66)	5.60 × 10^-^¹^0^	Largest effect; sparse target events
Cladribine	5/303	3,077/123,704	0.66 (0.27 to 1.61)	0.53 (0.22 to 1.29)	0.56 (0.21 to 1.19)	0.177	Insufficient information; not a protective finding

Adjusted estimates use Jeffreys-penalized logistic regression with contemporaneous active DMT comparators; BH adjustment applies to the five adjusted primary models. Each mechanism is a separate target-versus-pooled-reference contrast; the estimates are not a formal between-mechanism comparison.

aROR, adjusted reporting odds ratio; BH, Benjamini–Hochberg; CI, confidence interval; DMT, disease-modifying therapy; MH-ROR, Mantel–Haenszel reporting odds ratio; ROR, reporting odds ratio.

Tier A+B estimates were 0.89 (0.70–1.13) for anti-CD20 therapies, 1.79 (1.48–2.17) for S1P modulators, 1.20 (0.98–1.48) for natalizumab, 3.39 (2.41–4.78) for alemtuzumab, and 0.61 (0.28–1.32) for cladribine (**Supplementary Table S3**). Adding tier C events reduced the S1P and alemtuzumab estimates and changed the natalizumab estimate. Tier A remained the prespecified confirmatory endpoint; tier A+B+C was analyzed only as an exploratory sensitivity.

#### 3.2.1 Product-level decomposition of the S1P reporting signal

Fingolimod accounted for 53,117 of 60,450 S1P reports and 145 of 152 S1P tier A cases (95.4%) (**Figure 3A**; **Supplementary Table S5**). Its product-level active-comparator aROR was 2.15 (95% CI 1.75–2.64; multiplicity-adjusted *P* = 1.40 × 10^-^¹²). Siponimod had an aROR of 0.83 (0.38–1.82; six cases), and ozanimod had an aROR of 0.22 (0.05–1.10; one case). The ponesimod estimate was 1.49 (0.09–23.55), with no directly observed tier A case before penalization. All three estimates were information limited (**Figure 3B**). The four-product common-period test detected heterogeneity with a small effect size (*P* = 1.00 × 10^-5^; Cramér’s *V* = 0.063). Within the S1P class, fingolimod had greater reporting than newer products (aROR 10.59, 5.11–25.27). Because the reference contained only seven tier A events and failed the prespecified gate, this estimate remained exploratory.

**Figure 3:**
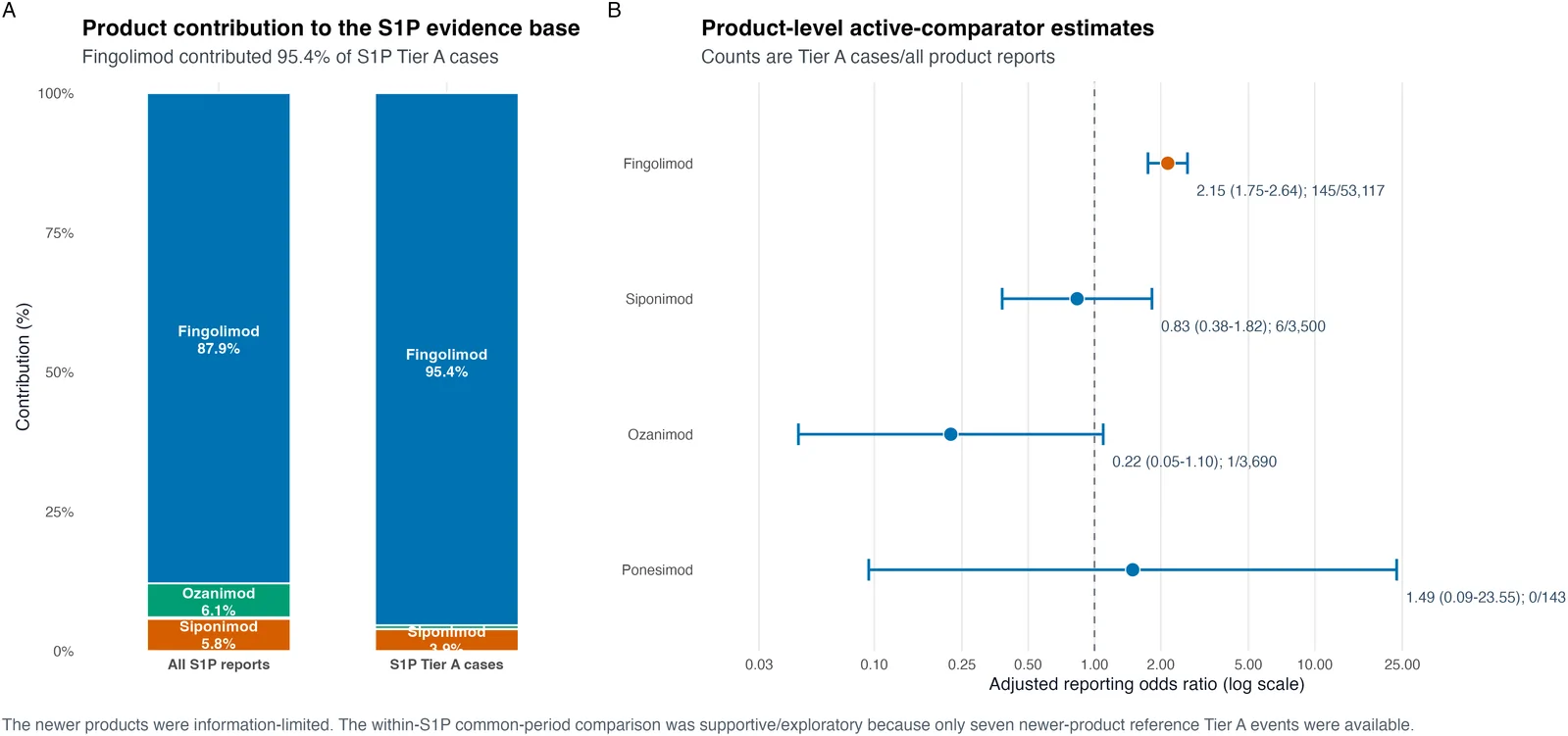
Product-level S1P results identify fingolimod as the signal driver. **(A)** Product contributions to all S1P reports and S1P tier A cases. **(B)** Product-specific aRORs and 95% CIs comparing each S1P product with contemporaneous non-S1P active DMT reports. The within-S1P common-period comparison was supportive and exploratory because the newer-product reference group contained only seven tier A events.

### 3.3 Pathogen composition showed mechanism-specific profiles

The adjusted global multinomial model included 523 tier A+B reports from the anti-CD20 core, S1P, natalizumab, and alemtuzumab groups. Cladribine was excluded because it contributed only six tier A+B reports. Pathogen composition differed across mechanisms in the adjusted multinomial model (likelihood-ratio statistic 210.59, 9 *df*; parametric bootstrap *P* = 9.99 × 10^-4^); the complementary unadjusted association had Cramér’s *V* = 0.415 (**Figure 4A**; **Supplementary Table S6**). All 1,000 bootstrap replicates were usable, and diagnostic checks identified no numerical instability. The four pathogen classes represented in this model were virus (235), fungus (105), bacterium (82), and other/mixed/unspecified (101).

**Figure 4:**
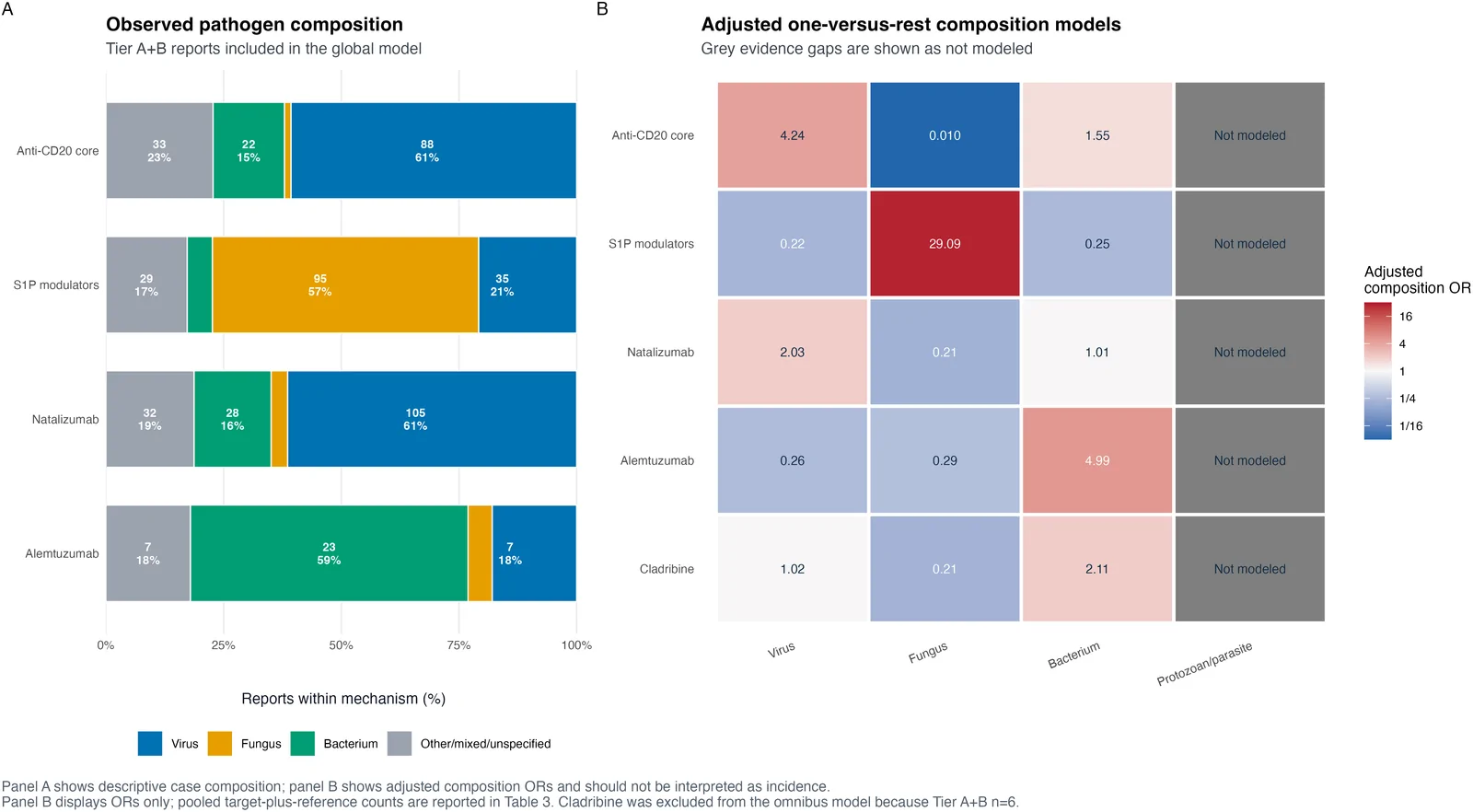
Mechanism-specific pathogen composition among tier A+B reports. **(A)** Observed pathogen-class composition by mechanism. **(B)** Adjusted one-versus-rest pathogen composition ORs. Gray cells failed the prespecified class-size gate. The adjusted global multinomial model detected heterogeneity (likelihood-ratio statistic 210.59, 9 *df*; bootstrap *P* = 9.99 × 10^-4^); the complementary unadjusted Cramér’s *V* was 0.415. These are reporting composition estimates, not incidence estimates.

Adjusted one-versus-rest models showed viral enrichment with anti-CD20 core therapies (composition OR 4.24, 95% CI 2.56–7.02; FDR-adjusted *P* = 7.86 × 10^-8^) (**Figure 4B**; **Table 3**). Natalizumab also showed viral enrichment (2.03, 1.26–3.26; *P* = 0.006). S1P modulators showed marked fungal enrichment (29.09, 15.77–53.64; *P* = 5.52 × 10^-^²^6^) and lower viral and bacterial composition odds. Bacterial reports were enriched with alemtuzumab (4.99, 2.27–11.01; *P* = 1.66 × 10^-4^). The estimates describe pathogen distribution among reported neuroinvasive infections, not infection incidence.

**Table 3:** Mechanism-specific pathogen composition among tier A+B reports.

DMT mechanism	Pathogen class	Pooled target-plus-reference analytic reports, *n*	Pooled pathogen-class reports, *n*	Adjusted composition OR (95% CI)	FDR-adjusted P	Model status
Anti-CD20 core	Virus	414	178	4.24 (2.56 to 7.02)	7.86 × 10^-8^	Modeled
Anti-CD20 core	Fungus	414	97	0.010 (0.003 to 0.041)	5.34 × 10^-^¹^0^	Modeled
Anti-CD20 core	Bacterium	414	68	1.55 (0.83 to 2.90)	0.231	Modeled
Anti-CD20 core	Protozoan/parasite	414	2	NE	NE	Not modeled: below binary downgrade threshold
S1P modulators	Virus	573	259	0.22 (0.14 to 0.35)	5.60 × 10^-^¹^0^	Modeled
S1P modulators	Fungus	573	114	29.09 (15.77 to 53.64)	5.52 × 10^-^²^6^	Modeled
S1P modulators	Bacterium	573	102	0.25 (0.13 to 0.49)	1.43 × 10^-4^	Modeled
S1P modulators	Protozoan/parasite	573	3	NE	NE	Not modeled: below binary downgrade threshold
Natalizumab	Virus	605	281	2.03 (1.26 to 3.26)	0.006	Modeled
Natalizumab	Fungus	605	114	0.21 (0.09 to 0.48)	6.23 × 10^-4^	Modeled
Natalizumab	Bacterium	605	105	1.01 (0.57 to 1.81)	0.980	Modeled
Natalizumab	Protozoan/parasite	605	5	NE	NE	Not modeled: below binary downgrade threshold
Alemtuzumab	Virus	504	213	0.26 (0.11 to 0.64)	0.006	Modeled
Alemtuzumab	Fungus	504	111	0.29 (0.08 to 1.11)	0.105	Modeled
Alemtuzumab	Bacterium	504	96	4.99 (2.27 to 11.01)	1.66 × 10^-4^	Modeled
Alemtuzumab	Protozoan/parasite	504	2	NE	NE	Not modeled: below binary downgrade threshold
Cladribine	Virus	284	135	1.02 (0.18 to 5.71)	0.980	Modeled
Cladribine	Fungus	284	71	0.21 (0.01 to 5.49)	0.439	Modeled
Cladribine	Bacterium	284	35	2.11 (0.27 to 16.54)	0.551	Modeled
Cladribine	Protozoan/parasite	284	0	NE	NE	Not modeled: below binary downgrade threshold

Composition ORs compare each pathogen class with all other pathogen classes among infection cases. Global adjusted multinomial heterogeneity: LRT = 210.59, *df* = 9, bootstrap *P* = 9.99 × 10^-4^. Sparse protozoan/parasite cells were retained descriptively but not assigned unstable one-versus-rest estimates.

CI, confidence interval; DMT, disease-modifying therapy; FDR, false discovery rate; OR, odds ratio.

### 3.4 Drug–pathogen association and CNS enrichment were not equivalent

The two drug–pathogen analyses addressed different questions (**Figure 5**; **Table 4**; **Supplementary Table S7**). In *post hoc* models comparing a specific CNS event with all other adverse events, all four prespecified pairs showed positive associations: anti-CD20–enterovirus aROR 11.38 (95% CI 4.09–31.66), S1P–*Cryptococcus* 59.74 (31.65–112.77), natalizumab–HSV/VZV 3.03 (2.16–4.25), and alemtuzumab–*Listeria* 92.22 (40.70–208.96). These event-specific estimates were especially sensitive to sparse counts and did not test whether a therapy preferentially shifted infection to the CNS.

**Figure 5:**
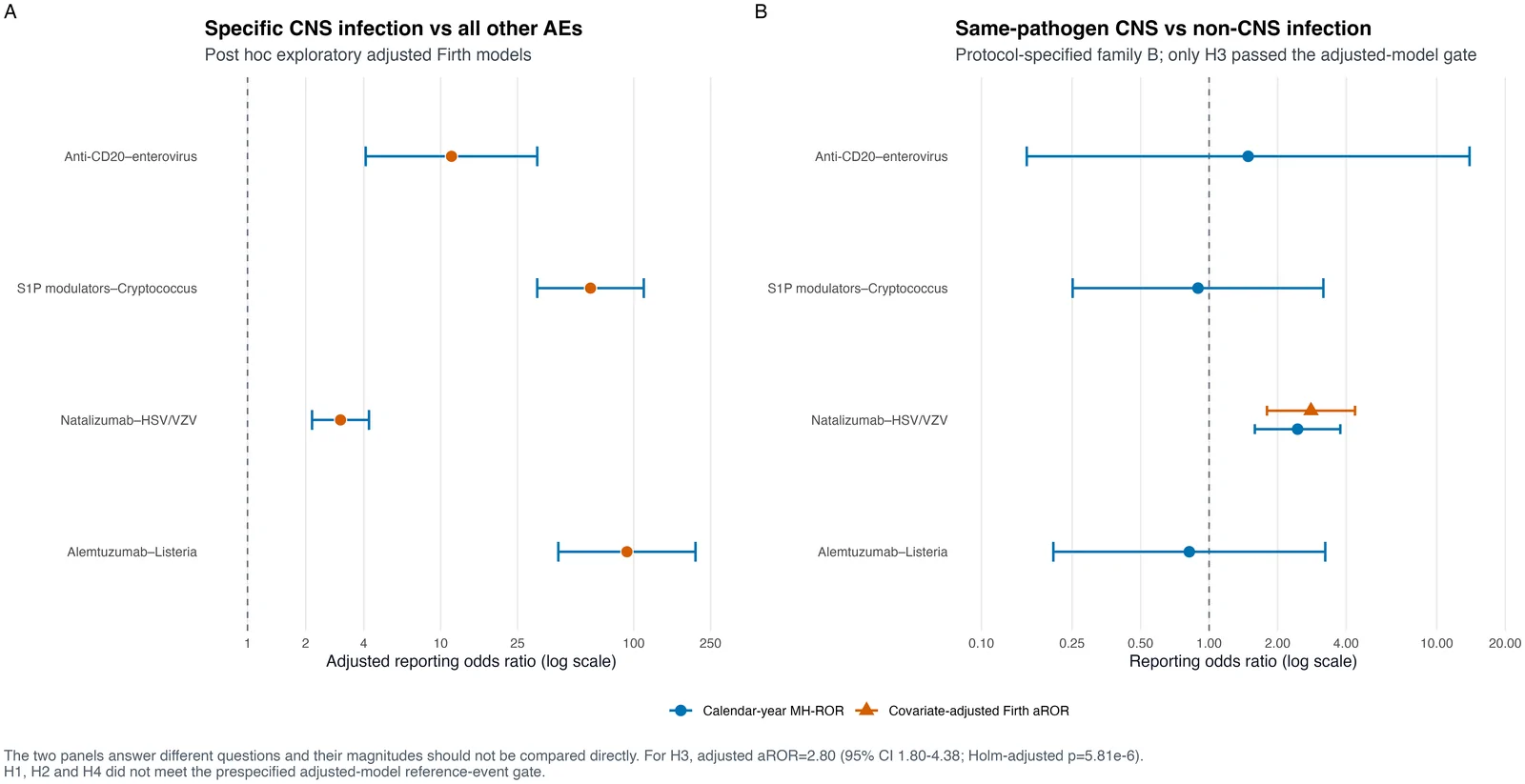
Drug–pathogen reporting associations did not uniformly indicate CNS enrichment. **(A)** *Post hoc* exploratory adjusted models comparing each specific CNS infection with all other adverse events. **(B)** Protocol-specified same-pathogen CNS-versus-non-CNS MH-RORs. H1 = anti-CD20–enterovirus, H2 = S1P–*Cryptococcus*, H3 = natalizumab–HSV/VZV, and H4 = alemtuzumab–*Listeria*. Only natalizumab–HSV/VZV passed the adjusted model reference-event gate; its adjusted aROR was 2.80 (95% CI 1.80–4.38; Holm-adjusted *P* = 5.81 × 10^-6^). The panels answer different questions, and their effect magnitudes should not be compared directly.

**Table 4:** Integrated drug–pathogen evidence across two distinct estimands.

Hypothesis	DMT mechanism	Pathogen	*Post hoc* target/reference CNS cases	Specific CNS infection vs. all other AEs, aROR (95% CI)	*Post hoc* multiplicity-adjusted *P*	Protocol target/reference CNS cases	Same-pathogen CNS vs. non-CNS, MH-ROR (95% CI)	Protocol Holm-adjusted *P*	Adjusted same-pathogen aROR (95% CI)	Adjusted Holm *P*	Integrated evidence
H1	Anti-CD20 core	Enterovirus	14/3	11.38 (4.09 to 31.66)	3.16 × 10^-6^	14/1	1.48 (0.16 to 13.93)	1.000	Not modeled (event gate not met)	NE	Event association; insufficient same-pathogen data for adjusted CNS-localization inference
H2	S1P modulators	*Cryptococcus*	91/9	59.74 (31.65 to 112.77)	6.75 × 10^-^³^6^	91/8	0.89 (0.25 to 3.18)	1.000	Not modeled (event gate not met)	NE	Strong event association; insufficient same-pathogen data for adjusted CNS-localization inference
H3	Natalizumab	HSV/VZV	62/91	3.03 (2.16 to 4.25)	2.97 × 10^-^¹^0^	62/78	2.45 (1.59 to 3.77)	0.003	2.80 (1.80 to 4.38)	5.81 × 10^-6^	Convergent event association and adjusted same-pathogen CNS enrichment
H4	Alemtuzumab	*Listeria*	23/6	92.22 (40.70 to 208.96)	6.59 × 10^-^²^7^	23/6	0.82 (0.21 to 3.24)	1.000	Not modeled (event gate not met)	NE	Strong sparse event association; insufficient same-pathogen data for adjusted CNS-localization inference

The *post hoc* all-AE estimand measures reporting association; the protocol-specified same-pathogen case–case estimand measures relative CNS enrichment. H1 = anti-CD20–enterovirus; H2 = S1P–*Cryptococcus*; H3 = natalizumab–HSV/VZV; H4 = alemtuzumab–*Listeria*. The two estimands should not be compared as the same effect.

AE, adverse event; aROR, adjusted reporting odds ratio; CI, confidence interval; CNS, central nervous system; DMT, disease-modifying therapy; MH-ROR, Mantel–Haenszel reporting odds ratio; NE, not estimable.

Only the natalizumab–HSV/VZV comparison passed the event gate for the protocol-specified adjusted same-pathogen model. Natalizumab–HSV/VZV had a calendar-year MH-ROR of 2.45 (1.59–3.77; Holm-adjusted *P* = 0.003) and an aROR of 2.80 (1.80–4.38; Holm-adjusted *P* = 5.81 × 10^-6^), indicating additional CNS enrichment. The anti-CD20–enterovirus, S1P–*Cryptococcus*, and alemtuzumab–*Listeria* estimates were imprecise and did not meet the adjusted model gate. Thus, positive event associations for these pairs were not accompanied by evidence of preferential CNS localization after conditioning on the same pathogen.

### 3.5 Sensitivity analyses and internal checks

Among 117 mechanism–scenario sensitivity estimates, 116 gated models converged (**Supplementary Figure S1**; **Supplementary Table S3**). Across the prespecified restrictions by reporter, seriousness, pandemic period, co-reported steroids or immunosuppressants, treatment carryover, and literature/study source, S1P and alemtuzumab point estimates were above 1 in converged models. Anti-CD20 estimates were at or below 1 with active comparators; natalizumab estimates varied by endpoint breadth and comparator, and cladribine models had few events with wide confidence intervals.

With interferon-beta/glatiramer acetate as the reference, aRORs were 6.25 (3.59–10.87) for anti-CD20 core therapies, 3.94 (2.88–5.39) for S1P modulators, 2.39 (1.84–3.09) for natalizumab, and 11.52 (6.96–19.08) for alemtuzumab (**Supplementary Figure S2**; **Supplementary Table S8**). With all other mapped MS DMTs as the reference, aRORs were 1.00 (0.78–1.29) for anti-CD20, 2.13 (1.75–2.58) for S1P, 1.50 (1.23–1.82) for natalizumab, 3.96 (2.77–5.66) for alemtuzumab, and 0.66 (0.28–1.52) for cladribine.

The cohort contained 10,384 coronavirus disease 2019 (COVID-19)/SARS-CoV-2 reports, most commonly with ocrelizumab, but only two overlapped tier A and nine overlapped tier A+B. After excluding all COVID-19/SARS-CoV-2 reports, tier A aRORs were 0.98 (0.77–1.26) for anti-CD20, 1.65 (1.35–2.01) for S1P, 1.27 (1.03–1.58) for natalizumab, 3.11 (2.17–4.46) for alemtuzumab, and 0.53 (0.23–1.24) for cladribine (**Supplementary Table S4**).

The natalizumab–PML MH-ROR was 15.48 (95% CI 13.60–17.63), and the S1P–herpes MH-ROR was 1.05 (95% CI 0.98–1.13) after calendar-year stratification (**Supplementary Figure S3**).

### 3.6 Serious outcomes and time to onset were descriptive

Report-level death outcome flags varied across exposure groups (**Table 1**): 7,649/145,084 (5.27%) for interferon-beta/glatiramer acetate, 917/42,794 (2.14%) for anti-CD20 core therapies, 107/3,203 (3.34%) for anti-CD20 extension products, 1,024/45,997 (2.23%) for the combined anti-CD20 groups, and 91/3,084 (2.95%) for alemtuzumab. These percentages were calculated across all reports, not among tier A+B infection cases; 7,629 of the 7,649 interferon-beta/glatiramer-acetate death reports were outside the tier A/B/C and PML/JCV outcome sets. Because FAERS lacks person-time, treatment duration, and reliable cause-of-death data, these fractions were treated as descriptive report characteristics and not as comparative mortality or case fatality estimates.

Neuroinvasive infection reports were highly selected for seriousness. Hospitalization and other serious outcomes were common, but proportions varied by product and were not treated as comparative clinical rates (**Supplementary Table S9**). TTO was available for 103 of 757 tier A+B reports. Compared with reports lacking usable dates, those with usable dates more often had known age (98.1% vs. 73.9%) and less often came from healthcare professionals (42.7% vs. 63.6%) (**Supplementary Figure S4**). Given this selection, TTO distributions were reported descriptively, without hazard, cumulative-incidence, or wear-out interpretation.

## 4 Discussion

Non-PML neuroinvasive infection reporting varied across MS-DMT mechanisms in 492,514 FAERS reports. S1P modulators and alemtuzumab had clear overall tier A signals, whereas the adjusted natalizumab signal was smaller. Anti-CD20 core therapies and cladribine showed no adjusted overall positive signal. Pathogen composition revealed a different pattern: viral reports were enriched with anti-CD20 therapies and natalizumab, fungal reports with S1P modulators, and bacterial reports with alemtuzumab. Drug–pathogen association also differed from CNS localization. Only natalizumab–HSV/VZV showed additional CNS enrichment in the same-pathogen analysis.

Population-based studies show that DMT safety patterns vary by outcome and healthcare setting and may not be fully captured during randomized trial follow-up (25). A Canadian study of infection-related healthcare use similarly found that treatment contrasts changed with severity and analytic definition (26). We addressed the narrower question of rare, non-PML neuroinvasive phenotypes. Reporting estimates from FAERS are therefore not numerically comparable with incidence estimates from population-based data.

The adjusted E1 estimate for anti-CD20 core therapy was compatible with no aggregate disproportionality for the high-certainty tier A endpoint (aROR 0.82, 95% CI 0.63–1.05; BH-adjusted *P* = 0.144) relative to contemporaneous active DMTs. It should not be interpreted as a protective effect or proof that clinically important risk is absent. Extended ocrelizumab trial data show generally stable serious infection rates (27, 28), whereas a meta-analysis of more than 19,000 anti-CD20-treated patients documented hypogammaglobulinemia and infection burden (29). In a real-world ocrelizumab cohort, treatment-emergent hypogammaglobulinemia was not associated with serious infection (30), and ofatumumab extension data showed low serious infection incidence with stable mean IgG over 3.5 years (31). These studies illustrate clinical heterogeneity but do not establish a susceptible subgroup in FAERS. E2 addressed a different estimand and showed higher viral composition odds among anti-CD20 tier A+B infection reports (OR 4.24, 95% CI 2.56–7.02), whereas the anti-CD20–enterovirus E3 comparison did not meet the prespecified reference-event gate. Any host factor heterogeneity requires denominator-based studies with prespecified interaction testing.

The pathogen composition result was more specific than the overall anti-CD20 estimate. Viral tier A+B reports were enriched, and the *post hoc* anti-CD20–enterovirus association was exploratory and sparse. B-cell depletion can impair maintenance of neutralizing antibodies, and disseminated enterovirus infection has occurred during maintenance ocrelizumab therapy (32). The same-pathogen analysis was too sparse to establish additional CNS enrichment. Enterovirus may be considered in a compatible clinical syndrome, particularly when humoral immune impairment is suspected; this finding does not justify routine screening or a change in anti-CD20 prescribing.

The S1P signal was robust but concentrated in fingolimod. A meta-analysis of 12 randomized trials found higher risks of overall and serious infection with fingolimod, including lower respiratory and herpesvirus infections (33). Postmarketing evidence is more specific for cryptococcal meningitis. A 60-case manufacturer safety-database series estimated a low absolute reporting rate but included serious and fatal outcomes (34). Clinical reports also show that cryptococcal disease can present subtly or resemble other neurological emergencies (35). Histoplasmosis has also been described, extending concern to invasive fungal disease in endemic settings and susceptible patients (36).

Fungal enrichment within reported neuroinvasive infections reduced the influence of the much larger pool of non-infectious adverse events. Fingolimod accounted for 95.4% of S1P tier A cases. A previous FAERS analysis also found product-level differences among S1P modulators, while emphasizing unequal marketing time and the limitations of spontaneous reports (37). S1P modulators differ in receptor selectivity and neuropharmacology, with fingolimod having the longest exposure history (38). Sparse data for siponimod, ozanimod, and ponesimod preclude comparative safety claims. The exploratory within-class aROR of 10.59 therefore localizes the observed signal but should not guide product selection.

The two *Cryptococcus* estimands conveyed different information. The S1P–*Cryptococcus* event association was large, whereas the same-pathogen CNS-versus-non-CNS estimate was imprecise and close to the null. Cryptococcal CNS disease was therefore disproportionately reported with S1P therapy, but preferential CNS localization was not demonstrated after conditioning on cryptococcal infection. During fingolimod treatment, persistent headache, cognitive change, subacute fever, cranial neuropathy, or unexplained intracranial hypertension should prompt consideration of cryptococcosis. FAERS cannot estimate the probability of that diagnosis.

Natalizumab produced the most consistent drug–pathogen result. VZV vasculopathy with retinitis has been reported during natalizumab therapy (39). An earlier case also raised concern about herpes simplex encephalitis when leukocyte trafficking into the CNS is impaired (40). A recent systematic review and meta-analysis synthesized clinically relevant HSV/VZV reactivation across MS DMTs, although direct comparative evidence remained limited (41).

Unlike systemic lymphocyte-depleting therapies, natalizumab directly reduces immune-cell entry across the blood–brain barrier through α4-integrin blockade. The positive event association and both same-pathogen estimates for H3 are therefore pharmacologically coherent. This result was more specific than the overall natalizumab estimate, which changed direction after adjustment and weakened when tier B was added. Compatible encephalitic, meningitic, vasculopathic, or retinal syndromes should prompt guideline-concordant CSF HSV/VZV testing and empiric antiviral treatment when clinically indicated. The analysis does not support routine antiviral prophylaxis. Confirmation requires data on treatment duration, previous therapy, monitoring, and clinical diagnostic detail.

Alemtuzumab had the largest overall tier A aROR and a marked bacterial composition signal. Long-term extension data show sustained efficacy alongside the need for prolonged monitoring after immune depletion and reconstitution (42). A multicenter real-world study reported frequent infections but no unexpected safety signal (43). The large FAERS estimate arose from 35 tier A reports and a therapy used in a selected population. Differential reporting of severe events therefore remains a substantial concern.

Prior clinical reports support the alemtuzumab–*Listeria* association. Invasive listeriosis has occurred soon after alemtuzumab infusion, prompting recommendations that food precautions begin before treatment (44). Two cases of *Listeria meningitis* within days of the first infusion cycle further suggest an acute post-depletion window (45). Our event-association result accords with this warning, but the same-pathogen analysis did not show additional CNS enrichment over non-CNS listeriosis. Prevention and early recognition of invasive disease remain the practical priorities. These include dietary counseling and prompt guideline-concordant assessment and empiric *Listeria*-active therapy when invasive infection is clinically suspected.

Cladribine contributed five tier A and six tier A+B reports. Its wide CIs and lack of a positive signal should not be interpreted as protection. In a multicenter Portuguese cohort, lymphocytopenia and infections were the most frequent adverse reactions, without a new safety signal (46). A four-center US study found that cladribine was prescribed to older patients with longer disease duration than participants in pivotal trials (47). The present data cannot exclude rare neuroinvasive infection. Larger postmarketing cohorts with exposure denominators are needed before a mechanism-specific conclusion can be drawn.

Interpretability depended on several design choices. We used the latest CASEID rather than PRIMARYID as the analysis unit and audited legacy identifier collisions. Products were aligned to contemporaneous comparators, missing covariates were retained as explicit categories, and outcome certainty was separated from breadth. Calendar-year control was consequential: the natalizumab estimate changed from below 1 in the crude analysis to above 1 after adjustment. Jeffreys-prior penalization reduced instability from sparse cells, while prespecified event gates prevented unsupported adjusted models. Positive controls, product decomposition, COVID-19 exclusion, carryover restrictions, comparator analyses, and the full sensitivity matrix identified where estimates were stable and where they were not.

The three estimands also needed to remain distinct. E1 measured neuroinvasive infection reporting against all other adverse events. E2 described pathogen composition among neuroinvasive infection reports, and E3 evaluated CNS localization among reports involving the same pathogen. The *Cryptococcus* and *Listeria* findings illustrate the consequence: a large event association can coexist with a null or imprecise localization estimate.

This study shares the limitations of spontaneous-reporting data. FAERS lacks a treated population denominator, person-time, systematic outcome confirmation, complete treatment duration, and reliable records of negative diagnostic tests. Reporting varies with disease severity, publicity, regulatory action, time on market, litigation, geography, reporter type, and clinical recognition. Standard deduplication may not remove every narrative concerning the same person. Coding may also fail to distinguish primary infection from reactivation, coinfection, immune-reconstitution phenomena, or an MS relapse initially considered infectious. Residual confounding by indication and previous DMT exposure is likely.

Disproportionality alone does not support causal inference. Causal claims require strong assumptions about confounding, underreporting, and the reporting probability of comparator events, which are rarely verifiable in FAERS (48). Published signals may also reflect incomplete validation and selective analytic choices (49). Multiple testing poses an additional problem in pharmacovigilance studies without an *a priori* hypothesis (50). We reduced these risks through locked outcome families, Holm or BH adjustment, global tests, and explicit labeling of *post hoc* analyses. False-positive and false-negative findings nevertheless remain possible.

Outcome misclassification is another concern. Tier A favored specificity and may have missed true cases coded with non-specific terms. Tier B depended on co-reported pathogen evidence, whereas tier C included clinically ambiguous syndromes and was excluded from confirmatory analyses. The full screening dictionary and its classification roles are provided in **Supplementary Data S1A**, **Supplementary Table S10**. The pathogen composition analysis was optimized for specificity rather than exhaustive ascertainment; infections without pathogen identification were not assigned to a named pathogen, and rare classes were underpowered for pathogen-specific inference. Expanding the endpoint to tier A+B+C increased event counts but changed several estimates, so it did not simply increase power. Data for cladribine and newer S1P products remained sparse. TTO availability was also strongly selected; onset and seriousness summaries therefore describe reports rather than comparative clinical outcomes.

The results support vigilance tailored to both treatment mechanism and pathogen. During anti-CD20 therapy, an unexplained chronic or disseminated viral syndrome may warrant assessment of humoral immunity and consideration of enterovirus testing when clinically compatible; this is not a recommendation for routine screening. Subacute meningitic or encephalitic symptoms during fingolimod treatment warrant early fungal evaluation, including cryptococcal antigen testing and appropriate CSF studies when clinically safe. Compatible HSV/VZV CNS syndromes during natalizumab therapy should prompt urgent, guideline-concordant CSF testing and empiric antiviral treatment when clinically indicated. Around alemtuzumab infusion, food precautions and guideline-concordant evaluation and treatment of suspected invasive listeriosis remain important.

These observations prioritize clinical questions rather than comparative treatment rules. Confirmation requires data that link treatment exposure and person-time to validated microbiology, CSF findings, imaging, and immune biomarkers. Multinational MS registries and linked claims–electronic health record data could estimate absolute and relative incidence for the four drug–pathogen pairs. Nested case–control studies could then examine treatment duration, previous DMTs, lymphocyte subsets, immunoglobulins, vaccination, and regional pathogen exposure. Future surveillance should continue to separate pathogen reporting from CNS localization.

## 5 Conclusion

Reporting of non-PML neuroinvasive infections varied descriptively across MS-DMT mechanisms in FAERS. The S1P signal was largely attributable to fingolimod and was characterized by fungal enrichment. Alemtuzumab had the largest overall tier A estimate and bacterial enrichment, whereas anti-CD20 therapies and natalizumab showed viral enrichment. Natalizumab–HSV/VZV was the only prespecified pair supported by both event-association and same-pathogen CNS-localization analyses. This pattern can guide clinical suspicion and the design of confirmatory studies, but these analyses neither quantify incidence nor establish causality.

## Data Availability

The original contributions presented in the study are included in the article/**Supplementary Material**. Further inquiries can be directed to the corresponding authors.
