# Supplementary material for: Non-PML neuroinvasive infections reported with multiple sclerosis disease-modifying therapies: a mechanism-based pharmacovigilance study, 2004–2025
Source: Front Immunol. 2026 Oct 13;17:1959164. doi: 10.3389/fimmu.2026.1959164 (PMC13710094; doi:10.3389/fimmu.2026.1959164)
Supplement: Supplementary file 1 [file DataSheet2.docx]

**Supplementary Material**

**Contents**

**Supplementary Tables**

Supplementary Table S1. MedDRA preferred-term dictionary for CNS outcome classification	**2**

Supplementary Table S2. Cohort construction, identifier, and sequential-treatment audit	**8**

Supplementary Table S3. Complete adjusted sensitivity analysis matrix	**9**

Supplementary Table S4. SARS-CoV-2 reporting audit and exclusion sensitivity	**14**

Supplementary Table S5. S1P product contribution and within-class heterogeneity	**16**

Supplementary Table S6. Full pathogen-composition models and global-model diagnostics	**17**

Supplementary Table S7. Four-cell data and adjusted same-pathogen CNS case-case models	**19**

Supplementary Table S8. Positive controls and alternative estimands/comparators	**20**

Supplementary Table S9. Serious outcomes, time-to-onset summaries, and availability diagnostics	**22**

Supplementary Table S10. Full screening dictionary audit and endpoint roles	27

**Supplementary Figures**

Supplementary Figure S1. Adjusted sensitivity and leave-one-drug-out matrix	**27**

Supplementary Figure S2. Bias-directed sensitivity analyses show comparator-dependent magnitude	**28**

Supplementary Figure S3. Internal validity checks and cross-scenario stability	**29**

Supplementary Figure S4. Time to onset is descriptive because date availability was selective	**30**

Supplementary Tables S1–S10 and Figures S1–S4 retain a fixed numbering scheme used consistently throughout the manuscript and Supplementary Material. Supplementary Table S10 is a dictionary-audit appendix, and Supplementary Data S1A provides the machine-readable full dictionary.

## Supplementary glossary and comparator definitions

Core denotes the locked strict-primary-suspect analysis group; Expanded denotes Tier A+B; Exploratory denotes Tier A+B+C; PS and SS denote primary-suspect and secondary-suspect drug roles; NE denotes not estimable; ROR denotes reporting odds ratio; MH-ROR denotes the calendar-year Mantel–Haenszel ROR; aROR denotes covariate-adjusted ROR; BH and Holm denote the corresponding multiplicity adjustments. Profile-likelihood CIs are reported for locked primary penalized models and Wald CIs for other sensitivity models. For adjusted Firth models, the general event gate was at least 30 total outcome events and 10 reference events; the E3 same-pathogen gate additionally required at least five CNS and five non-CNS reports in each comparison. P values are shown to three decimal places when P≥0.001 and in scientific notation with three significant digits when P<0.001. ‘Contemporaneous active DMTs’ denotes reports from the target's launch quarter onward in all other primary-mechanism or immune-other DMT groups, excluding interferon-beta/glatiramer acetate and anti-CD20 extension-only products. ‘All other mapped MS DMTs’ denotes all mapped non-target DMT reports in the same launch-defined window, including interferon-beta/glatiramer acetate and extension products. The strong-immunosuppressant/antineoplastic exclusion removed reports co-reporting cyclophosphamide, methotrexate, azathioprine, mycophenolate, tacrolimus, cyclosporine/ciclosporin, sirolimus, everolimus, temsirolimus, chlorambucil, fludarabine, bendamustine, doxorubicin, vincristine, bleomycin, cisplatin, carboplatin, pemetrexed, or gemcitabine.

# Supplementary Table S1. MedDRA preferred-term dictionary for CNS outcome classification

MedDRA version 29.0. Supplementary Table S1 lists the 163 terms that directly determine CNS tier or explicit exclusion; the remaining 1,485 terms in the complete 1,648-term screening dictionary are provided as Supplementary Data S1A. Explicit exclusions take precedence over A1/A2/C classification in the locked classifier.

| **MedDRA version** | **PT code** | **Preferred term (English)** | **Dictionary tier** | **Classification role** | **Pathogen class** | **Specific pathogen** | **Confirmatory hypothesis** |
| --- | --- | --- | --- | --- | --- | --- | --- |
| 29 | 10080441 | Adenoviral encephalitis | A1 | Tier A direct definition (A1) | virus | adenovirus |  |
| 29 | 10080442 | Adenoviral meningitis | A1 | Tier A direct definition (A1) | virus | adenovirus |  |
| 29 | 10001984 | Amoebic brain abscess | A1 | Tier A direct definition (A1) | Protozoan parasite |  |  |
| 29 | 10090884 | Central nervous system bacterial infection | A1 | Tier A direct definition (A1) | bacterium |  |  |
| 29 | 10061035 | Central nervous system enteroviral infection | A1 | Tier A direct definition (A1) | virus | enterovirus |  |
| 29 | 10072805 | Central nervous system fungal infection | A1 | Tier A direct definition (A1) | fungus |  |  |
| 29 | 10090002 | Central nervous system listeria infection | A1 | Tier A direct definition (A1) | bacterium | listeria | H4 |
| 29 | 10061037 | Central nervous system viral infection | A1 | Tier A direct definition (A1) | virus |  |  |
| 29 | 10051597 | Cerebral aspergillosis | A1 | Tier A direct definition (A1) | fungus | aspergillus |  |
| 29 | 10057854 | Cerebral toxoplasmosis | A1 | Tier A direct definition (A1) | Protozoan parasite | toxoplasma |  |
| 29 | 10008761 | Choriomeningitis lymphocytic | A1 | Tier A direct definition (A1) | virus |  |  |
| 29 | 10054214 | Coccidioides encephalitis | A1 | Tier A direct definition (A1) | fungus | coccidioides |  |
| 29 | 10084824 | Cryptococcal meningoencephalitis | A1 | Tier A direct definition (A1) | fungus | cryptococcus | H2 |
| 29 | 10065621 | Cytomegalovirus myelomeningoradiculitis | A1 | Tier A direct definition (A1) | virus | cmv |  |
| 29 | 10014596 | Encephalitis Japanese B | A1 | Tier A direct definition (A1) | virus |  |  |
| 29 | 10014583 | Encephalitis australia | A1 | Tier A direct definition (A1) | virus |  |  |
| 29 | 10014584 | Encephalitis california | A1 | Tier A direct definition (A1) | virus |  |  |
| 29 | 10014586 | Encephalitis cytomegalovirus | A1 | Tier A direct definition (A1) | virus | cmv |  |
| 29 | 10014587 | Encephalitis eastern equine | A1 | Tier A direct definition (A1) | virus |  |  |
| 29 | 10063946 | Encephalitis enteroviral | A1 | Tier A direct definition (A1) | virus | enterovirus | H1 |
| 29 | 10014590 | Encephalitis herpes | A1 | Tier A direct definition (A1) | virus | hsv | H3 |
| 29 | 10058094 | Encephalitis influenzal | A1 | Tier A direct definition (A1) | virus |  |  |
| 29 | 10014597 | Encephalitis meningococcal | A1 | Tier A direct definition (A1) | bacterium | meningococcus |  |
| 29 | 10014598 | Encephalitis mumps | A1 | Tier A direct definition (A1) | virus |  |  |
| 29 | 10061119 | Encephalitis rickettsial | A1 | Tier A direct definition (A1) | bacterium |  |  |
| 29 | 10014611 | Encephalitis venezuelan equine | A1 | Tier A direct definition (A1) | virus |  |  |
| 29 | 10014614 | Encephalitis western equine | A1 | Tier A direct definition (A1) | virus |  |  |
| 29 | 10014622 | Encephalomyelitis rubella | A1 | Tier A direct definition (A1) | virus |  |  |
| 29 | 10083411 | HIV meningoencephalitis | A1 | Tier A direct definition (A1) | virus |  |  |
| 29 | 10019953 | Herpes simplex encephalitis | A1 | Tier A direct definition (A1) | virus | hsv | H3 |
| 29 | 10019956 | Herpes simplex meningitis | A1 | Tier A direct definition (A1) | virus | hsv | H3 |
| 29 | 10074247 | Herpes simplex meningoencephalitis | A1 | Tier A direct definition (A1) | virus | hsv | H3 |
| 29 | 10074250 | Herpes simplex meningomyelitis | A1 | Tier A direct definition (A1) | virus | hsv | H3 |
| 29 | 10074259 | Herpes zoster meningitis | A1 | Tier A direct definition (A1) | virus | vzv | H3 |
| 29 | 10074248 | Herpes zoster meningoencephalitis | A1 | Tier A direct definition (A1) | virus | vzv | H3 |
| 29 | 10074251 | Herpes zoster meningomyelitis | A1 | Tier A direct definition (A1) | virus | vzv | H3 |
| 29 | 10079327 | Herpes zoster meningoradiculitis | A1 | Tier A direct definition (A1) | virus | vzv | H3 |
| 29 | 10081897 | Human herpesvirus 6 encephalitis | A1 | Tier A direct definition (A1) | virus | hhv6 |  |
| 29 | 10086521 | Jamestown Canyon encephalitis | A1 | Tier A direct definition (A1) | virus |  |  |
| 29 | 10054116 | Listeria encephalitis | A1 | Tier A direct definition (A1) | bacterium | listeria | H4 |
| 29 | 10080466 | Measles meningitis | A1 | Tier A direct definition (A1) | virus |  |  |
| 29 | 10084029 | Meningitis Escherichia | A1 | Tier A direct definition (A1) | bacterium |  |  |
| 29 | 10073245 | Meningitis aspergillus | A1 | Tier A direct definition (A1) | fungus | aspergillus |  |
| 29 | 10049078 | Meningitis borrelia | A1 | Tier A direct definition (A1) | bacterium | borrelia |  |
| 29 | 10027205 | Meningitis candida | A1 | Tier A direct definition (A1) | fungus | candida |  |
| 29 | 10027207 | Meningitis coccidioides | A1 | Tier A direct definition (A1) | fungus | coccidioides |  |
| 29 | 10027208 | Meningitis coxsackie viral | A1 | Tier A direct definition (A1) | virus | enterovirus |  |
| 29 | 10069856 | Meningitis cronobacter | A1 | Tier A direct definition (A1) | bacterium |  |  |
| 29 | 10027209 | Meningitis cryptococcal | A1 | Tier A direct definition (A1) | fungus | cryptococcus | H2 |
| 29 | 10027231 | Meningitis echo viral | A1 | Tier A direct definition (A1) | virus | enterovirus |  |
| 29 | 10027232 | Meningitis enterococcal | A1 | Tier A direct definition (A1) | bacterium |  |  |
| 29 | 10027233 | Meningitis enteroviral | A1 | Tier A direct definition (A1) | virus | enterovirus |  |
| 29 | 10073246 | Meningitis exserohilum | A1 | Tier A direct definition (A1) | fungus |  |  |
| 29 | 10027239 | Meningitis gonococcal | A1 | Tier A direct definition (A1) | bacterium |  |  |
| 29 | 10027241 | Meningitis haemophilus | A1 | Tier A direct definition (A1) | bacterium |  |  |
| 29 | 10027242 | Meningitis herpes | A1 | Tier A direct definition (A1) | virus | hsv | H3 |
| 29 | 10027243 | Meningitis histoplasma | A1 | Tier A direct definition (A1) | fungus | histoplasma |  |
| 29 | 10027247 | Meningitis leptospiral | A1 | Tier A direct definition (A1) | bacterium |  |  |
| 29 | 10027248 | Meningitis listeria | A1 | Tier A direct definition (A1) | bacterium | listeria | H4 |
| 29 | 10027249 | Meningitis meningococcal | A1 | Tier A direct definition (A1) | bacterium | meningococcus |  |
| 29 | 10027250 | Meningitis mumps | A1 | Tier A direct definition (A1) | virus |  |  |
| 29 | 10027253 | Meningitis pneumococcal | A1 | Tier A direct definition (A1) | bacterium | pneumococcus |  |
| 29 | 10027254 | Meningitis salmonella | A1 | Tier A direct definition (A1) | bacterium |  |  |
| 29 | 10027255 | Meningitis staphylococcal | A1 | Tier A direct definition (A1) | bacterium | staphylococcus |  |
| 29 | 10027256 | Meningitis streptococcal | A1 | Tier A direct definition (A1) | bacterium | streptococcus |  |
| 29 | 10048848 | Meningitis toxoplasmal | A1 | Tier A direct definition (A1) | Protozoan parasite | toxoplasma |  |
| 29 | 10027258 | Meningitis trypanosomal | A1 | Tier A direct definition (A1) | Protozoan parasite |  |  |
| 29 | 10027259 | Meningitis tuberculous | A1 | Tier A direct definition (A1) | bacterium | tuberculosis |  |
| 29 | 10063032 | Meningoencephalitis amoebic | A1 | Tier A direct definition (A1) | Protozoan parasite |  |  |
| 29 | 10065171 | Meningoencephalitis helminthic | A1 | Tier A direct definition (A1) | Protozoan parasite |  |  |
| 29 | 10053586 | Meningoencephalitis herpes simplex neonatal | A1 | Tier A direct definition (A1) | virus | hsv | H3 |
| 29 | 10074249 | Meningomyelitis herpes | A1 | Tier A direct definition (A1) | virus | hsv | H3 |
| 29 | 10053981 | Murray Valley encephalitis | A1 | Tier A direct definition (A1) | virus |  |  |
| 29 | 10052057 | Neuroborreliosis | A1 | Tier A direct definition (A1) | bacterium | borrelia |  |
| 29 | 10029339 | Neurosyphilis | A1 | Tier A direct definition (A1) | bacterium |  |  |
| 29 | 10080816 | Phaeohyphomycotic brain abscess | A1 | Tier A direct definition (A1) | fungus |  |  |
| 29 | 10036011 | Polioencephalitis | A1 | Tier A direct definition (A1) | virus | enterovirus | H1 |
| 29 | 10091534 | Powassan virus encephalitis | A1 | Tier A direct definition (A1) | virus |  |  |
| 29 | 10074185 | Pseudomonas aeruginosa meningitis | A1 | Tier A direct definition (A1) | bacterium |  |  |
| 29 | 10041896 | St. Louis encephalitis | A1 | Tier A direct definition (A1) | virus |  |  |
| 29 | 10042297 | Subacute sclerosing panencephalitis | A1 | Tier A direct definition (A1) | virus |  |  |
| 29 | 10043847 | Tick-borne viral encephalitis | A1 | Tier A direct definition (A1) | virus |  |  |
| 29 | 10086746 | Varicella encephalitis | A1 | Tier A direct definition (A1) | virus | vzv | H3 |
| 29 | 10086747 | Varicella meningitis | A1 | Tier A direct definition (A1) | virus | vzv | H3 |
| 29 | 10080431 | Yersinia meningitis | A1 | Tier A direct definition (A1) | bacterium |  |  |
| 29 | 10006105 | Brain abscess | A2 | Tier A direct definition (A2) | unspecified |  |  |
| 29 | 10074682 | Brain empyema | A2 | Tier A direct definition (A2) | unspecified |  |  |
| 29 | 10009719 | CNS ventriculitis | A2 | Tier A direct definition (A2) | unspecified |  |  |
| 29 | 10061044 | Central nervous system abscess | A2 | Tier A direct definition (A2) | unspecified |  |  |
| 29 | 10061036 | Central nervous system infection | A2 | Tier A direct definition (A2) | unspecified |  |  |
| 29 | 10013874 | Dural abscess | A2 | Tier A direct definition (A2) | unspecified |  |  |
| 29 | 10065185 | Encephalitis bacterial | A2 | Tier A direct definition (A2) | bacterium |  |  |
| 29 | 10065170 | Encephalitis fungal | A2 | Tier A direct definition (A2) | fungus |  |  |
| 29 | 10061118 | Encephalitis protozoal | A2 | Tier A direct definition (A2) | Protozoan parasite |  |  |
| 29 | 10014612 | Encephalitis viral | A2 | Tier A direct definition (A2) | virus |  |  |
| 29 | 10086129 | Encephalomyelitis bacterial | A2 | Tier A direct definition (A2) | bacterium |  |  |
| 29 | 10086128 | Encephalomyelitis viral | A2 | Tier A direct definition (A2) | virus |  |  |
| 29 | 10014965 | Ependymitis | A2 | Tier A direct definition (A2) | unspecified |  |  |
| 29 | 10061846 | Extradural abscess | A2 | Tier A direct definition (A2) | unspecified |  |  |
| 29 | 10082062 | Intracranial infection | A2 | Tier A direct definition (A2) | unspecified |  |  |
| 29 | 10027202 | Meningitis bacterial | A2 | Tier A direct definition (A2) | bacterium |  |  |
| 29 | 10027236 | Meningitis fungal | A2 | Tier A direct definition (A2) | fungus |  |  |
| 29 | 10058780 | Meningitis neonatal | A2 | Tier A direct definition (A2) | unspecified |  |  |
| 29 | 10027260 | Meningitis viral | A2 | Tier A direct definition (A2) | virus |  |  |
| 29 | 10074672 | Meningoencephalitis viral | A2 | Tier A direct definition (A2) | virus |  |  |
| 29 | 10069588 | Parasitic encephalitis | A2 | Tier A direct definition (A2) | Protozoan parasite |  |  |
| 29 | 10088135 | Post procedural meningitis | A2 | Tier A direct definition (A2) | unspecified |  |  |
| 29 | 10076050 | Spinal cord abscess | A2 | Tier A direct definition (A2) | unspecified |  |  |
| 29 | 10049654 | Spinal cord infection | A2 | Tier A direct definition (A2) | unspecified |  |  |
| 29 | 10075486 | Spinal empyema | A2 | Tier A direct definition (A2) | unspecified |  |  |
| 29 | 10068885 | Subarachnoid abscess | A2 | Tier A direct definition (A2) | unspecified |  |  |
| 29 | 10083083 | Subdural abscess | A2 | Tier A direct definition (A2) | unspecified |  |  |
| 29 | 10065765 | Cranial nerve infection | C | Tier C exploratory |  |  |  |
| 29 | 10014581 | Encephalitis | C | Tier C exploratory |  |  |  |
| 29 | 10048997 | Encephalitis brain stem | C | Tier C exploratory |  |  |  |
| 29 | 10052369 | Encephalitis lethargica | C | Tier C exploratory |  |  |  |
| 29 | 10014619 | Encephalomyelitis | C | Tier C exploratory |  |  |  |
| 29 | 10027199 | Meningitis | C | Tier C exploratory |  |  |  |
| 29 | 10027201 | Meningitis aseptic | C | Tier C exploratory |  |  |  |
| 29 | 10028524 | Myelitis | C | Tier C exploratory |  |  |  |
| 29 | 10051295 | Neurological infection | C | Tier C exploratory |  |  |  |
| 29 | 10056332 | Panencephalitis | C | Tier C exploratory |  |  |  |
| 29 | 10000709 | Acute disseminated encephalomyelitis | Exclude: non-infectious | Excluded non-infectious mimic |  |  |  |
| 29 | 10063063 | Appendicitis noninfective | Exclude: non-infectious | Excluded non-infectious mimic |  |  |  |
| 29 | 10075691 | Autoimmune encephalopathy | Exclude: non-infectious | Excluded non-infectious mimic |  |  |  |
| 29 | 10011417 | Croup noninfectious | Exclude: non-infectious | Excluded non-infectious mimic |  |  |  |
| 29 | 10063057 | Cystitis noninfective | Exclude: non-infectious | Excluded non-infectious mimic |  |  |  |
| 29 | 10056387 | Encephalitis allergic | Exclude: non-infectious | Excluded non-infectious mimic |  |  |  |
| 29 | 10072378 | Encephalitis autoimmune | Exclude: non-infectious | Excluded non-infectious mimic |  |  |  |
| 29 | 10014602 | Encephalitis post immunisation | Exclude: non-infectious | Excluded non-infectious mimic |  |  |  |
| 29 | 10014607 | Encephalitis toxic | Exclude: non-infectious | Excluded non-infectious mimic |  |  |  |
| 29 | 10062608 | Endocarditis noninfective | Exclude: non-infectious | Excluded non-infectious mimic |  |  |  |
| 29 | 10083074 | Immune-mediated encephalitis | Exclude: non-infectious | Excluded non-infectious mimic |  |  |  |
| 29 | 10084701 | Immune-mediated encephalopathy | Exclude: non-infectious | Excluded non-infectious mimic |  |  |  |
| 29 | 10088960 | Immune-mediated myelitis | Exclude: non-infectious | Excluded non-infectious mimic |  |  |  |
| 29 | 10027206 | Meningitis chemical | Exclude: non-infectious | Excluded non-infectious mimic |  |  |  |
| 29 | 10058760 | Meningitis noninfective | Exclude: non-infectious | Excluded non-infectious mimic |  |  |  |
| 29 | 10071764 | Noninfectious myelitis | Exclude: non-infectious | Excluded non-infectious mimic |  |  |  |
| 29 | 10071648 | Noninfectious peritonitis | Exclude: non-infectious | Excluded non-infectious mimic |  |  |  |
| 29 | 10082151 | Noninfectious urinary meatitis | Exclude: non-infectious | Excluded non-infectious mimic |  |  |  |
| 29 | 10076775 | Noninfective bartholinitis | Exclude: non-infectious | Excluded non-infectious mimic |  |  |  |
| 29 | 10062644 | Noninfective bronchitis | Exclude: non-infectious | Excluded non-infectious mimic |  |  |  |
| 29 | 10074696 | Noninfective chorioretinitis | Exclude: non-infectious | Excluded non-infectious mimic |  |  |  |
| 29 | 10074701 | Noninfective conjunctivitis | Exclude: non-infectious | Excluded non-infectious mimic |  |  |  |
| 29 | 10074712 | Noninfective encephalitis | Exclude: non-infectious | Excluded non-infectious mimic |  |  |  |
| 29 | 10074713 | Noninfective encephalomyelitis | Exclude: non-infectious | Excluded non-infectious mimic |  |  |  |
| 29 | 10074706 | Noninfective epididymitis | Exclude: non-infectious | Excluded non-infectious mimic |  |  |  |
| 29 | 10074863 | Noninfective gingivitis | Exclude: non-infectious | Excluded non-infectious mimic |  |  |  |
| 29 | 10086116 | Noninfective mastoiditis | Exclude: non-infectious | Excluded non-infectious mimic |  |  |  |
| 29 | 10078830 | Noninfective myringitis | Exclude: non-infectious | Excluded non-infectious mimic |  |  |  |
| 29 | 10074436 | Noninfective oophoritis | Exclude: non-infectious | Excluded non-infectious mimic |  |  |  |
| 29 | 10074699 | Noninfective retinitis | Exclude: non-infectious | Excluded non-infectious mimic |  |  |  |
| 29 | 10075243 | Noninfective sialoadenitis | Exclude: non-infectious | Excluded non-infectious mimic |  |  |  |
| 29 | 10058461 | Orchitis noninfective | Exclude: non-infectious | Excluded non-infectious mimic |  |  |  |
| 29 | 10071066 | Posterior reversible encephalopathy syndrome | Exclude: non-infectious | Excluded non-infectious mimic |  |  |  |
| 29 | 10062903 | Urethritis noninfective | Exclude: non-infectious | Excluded non-infectious mimic |  |  |  |
| 29 | 10075613 | Anti-JC virus antibody index | Exclude: PML/JCV | Excluded PML/JCV outcome |  |  |  |
| 29 | 10092894 | Anti-JC virus antibody positive | Exclude: PML/JCV | Excluded PML/JCV outcome |  |  |  |
| 29 | 10078957 | JC virus CSF test positive | Exclude: PML/JCV | Excluded PML/JCV outcome |  |  |  |
| 29 | 10074361 | JC virus granule cell neuronopathy | Exclude: PML/JCV | Excluded PML/JCV outcome | virus |  |  |
| 29 | 10023163 | JC virus infection | Exclude: PML/JCV | Excluded PML/JCV outcome | virus |  |  |
| 29 | 10036807 | Progressive multifocal leukoencephalopathy | Exclude: PML/JCV | Excluded PML/JCV outcome | virus |  |  |
| 29 | 10092834 | Progressive multifocal leukoencephalopathy associated immune reconstitution inflammatory syndrome | Exclude: PML/JCV | Excluded PML/JCV outcome | virus |  |  |

# Supplementary Table S2. Cohort construction, identifier, and sequential-treatment audit

The audit preserves cohort-flow counts, reused historical AERS identifiers, unique-primary-suspect checks, and date-limited carryover exclusions.

| **Section** | **Item/mechanism** | **Reports** | **Tier A cases** | **Other MS-DMT reported** | **THER date available** | **Carryover exclusions** | **Carryover Tier A** | **Note** |
| --- | --- | --- | --- | --- | --- | --- | --- | --- |
| Cohort flow | Any-version DMT PRIMARYIDs | 1407520 |  |  |  |  |  | Sequential cohort construction |
| Cohort flow | Candidate CASEIDs | 1085600 |  |  |  |  |  | Sequential cohort construction |
| Cohort flow | Latest deduplicated CASEIDs | 1073073 |  |  |  |  |  | Sequential cohort construction |
| Cohort flow | Deleted cases removed | 12527 |  |  |  |  |  | Sequential cohort construction |
| Cohort flow | Latest reports with mapped DMT | 1072728 |  |  |  |  |  | Sequential cohort construction |
| Cohort flow | DRUG_SEQ-linked MS exposures | 682345 |  |  |  |  |  | Sequential cohort construction |
| Cohort flow | Strict PS/no other DMT PS-SS/eligible launch/no exclusion (A3) | 492514 |  |  |  |  |  | Sequential cohort construction |
| Identifier audit | Legacy PRIMARYID values reused across quarters | 54 |  |  |  |  |  | Identifier collision can cross-link DRUG/INDI/REAC rows when using PRIMARYID alone |
| Identifier audit | Deduplicated reports excluded from PS+SS role sensitivity | 108 |  |  |  |  |  | Conservative data-quality exclusion limited to the role-misclassification sensitivity |
| Sequential-treatment audit | Interferon-beta/glatiramer acetate | 145084 | 123 | 912 | 117 | 0 | 0 | THER available among other-DMT reports: 12.8%; sequence-time uncertain: 286 |
| Sequential-treatment audit | Other active/immune DMT | 102723 | 68 | 2760 | 5 | 0 | 0 | THER available among other-DMT reports: 0.2%; sequence-time uncertain: 776 |
| Sequential-treatment audit | Anti-CD20 extension | 3203 | 11 | 29 | 0 | 0 | 0 | THER available among other-DMT reports: 0.0%; sequence-time uncertain: 17 |
| Sequential-treatment audit | Natalizumab | 132099 | 159 | 15555 | 4617 | 2 | 0 | THER available among other-DMT reports: 29.7%; sequence-time uncertain: 147 |
| Sequential-treatment audit | S1P modulators | 60450 | 152 | 209 | 3 | 0 | 0 | THER available among other-DMT reports: 1.4%; sequence-time uncertain: 84 |
| Sequential-treatment audit | Alemtuzumab | 3084 | 35 | 37 | 0 | 0 | 0 | THER available among other-DMT reports: 0.0%; sequence-time uncertain: 24 |
| Sequential-treatment audit | Anti-CD20 core | 42794 | 122 | 152 | 0 | 0 | 0 | THER available among other-DMT reports: 0.0%; sequence-time uncertain: 68 |
| Sequential-treatment audit | Cladribine | 3077 | 5 | 1 | 0 | 0 | 0 | THER available among other-DMT reports: 0.0%; sequence-time uncertain: 0 |

# Supplementary Table S3. Complete adjusted sensitivity analysis matrix

All 117 specifications are reported regardless of direction or statistical significance; 116 met the event gate and converged. Core=locked strict-PS Tier A; Expanded=Tier A+B; Exploratory=Tier A+B+C; PS/SS=primary-/secondary-suspect. The contemporaneous active-DMT reference excludes interferon-beta/glatiramer acetate and anti-CD20 extension products. Locked-primary rows reproduce penalized profile-likelihood CIs; other sensitivity rows use Wald CIs.

|  | **Analysis tier** | **DMT mechanism** | **Outcome** | **Comparator** | **Target cases** | **Target non-cases** | **Reference cases** | **Reference non-cases** | **Target reports** | **Reference reports** | **Adjusted Firth aROR (95% CI; profile for locked primary, Wald otherwise)** | **Nominal P** | **Within-scenario BH P** | **CI status** | **Model gate** | **Converged** | **Period** | **Specification note** |
| --- | --- | --- | --- | --- | --- | --- | --- | --- | --- | --- | --- | --- | --- | --- | --- | --- | --- | --- |
| Locked primary Tier A | Core | Anti-CD20 core | Tier A | Contemporaneous active DMTs | 122 | 42672 | 253 | 127362 | 42794 | 127615 | 0.82 (0.63 to 1.05) | **0.115** | 0.144 | 95% CI includes 1 | Met | Yes | 2017Q2 to 2025Q4 | Locked primary analysis |
| Locked primary Tier A | Core | S1P modulators | Tier A | Contemporaneous active DMTs | 152 | 60298 | 367 | 267168 | 60450 | 267535 | 1.77 (1.44 to 2.16) | 2.13 × 10⁻⁸ | 5.32 × 10⁻⁸ | 95% CI above 1 | Met | Yes | 2010Q4 to 2025Q4 | Locked primary analysis |
| Locked primary Tier A | Core | Natalizumab | Tier A | Contemporaneous active DMTs | 159 | 131940 | 382 | 211578 | 132099 | 211960 | 1.32 (1.06 to 1.64) | 0.012 | 0.020 | 95% CI above 1 | Met | Yes | 2006Q2 to 2025Q4 | Locked primary analysis |
| Locked primary Tier A | Core | Alemtuzumab | Tier A | Contemporaneous active DMTs | 35 | 3049 | 418 | 218400 | 3084 | 218818 | 3.28 (2.24 to 4.66) | 1.12 × 10⁻¹⁰ | 5.60 × 10⁻¹⁰ | 95% CI above 1 | Met | Yes | 2014Q4 to 2025Q4 | Locked primary analysis |
| Locked primary Tier A | Core | Cladribine | Tier A | Contemporaneous active DMTs | 5 | 3072 | 303 | 123401 | 3077 | 123704 | 0.56 (0.21 to 1.19) | 0.177 | 0.177 | 95% CI includes 1 | Met | Yes | 2019Q2 to 2025Q4 | Locked primary analysis |
| Expanded Tier A+B | Core | Anti-CD20 core | Tier A+B | Contemporaneous active DMTs | 145 | 42649 | 274 | 127341 | 42794 | 127615 | 0.89 (0.70 to 1.13) | 0.325 | 0.325 | 95% CI includes 1 | Met | Yes | 2017Q2 to 2025Q4 | Expanded outcome; Tier A plus Tier B |
| Expanded Tier A+B | Core | S1P modulators | Tier A+B | Contemporaneous active DMTs | 168 | 60282 | 411 | 267124 | 60450 | 267535 | 1.79 (1.48 to 2.17) | 1.51 × 10⁻⁹ | 3.78 × 10⁻⁹ | 95% CI above 1 | Met | Yes | 2010Q4 to 2025Q4 | Expanded outcome; Tier A plus Tier B |
| Expanded Tier A+B | Core | Natalizumab | Tier A+B | Contemporaneous active DMTs | 171 | 131928 | 434 | 211526 | 132099 | 211960 | 1.20 (0.98 to 1.48) | 0.082 | 0.136 | 95% CI includes 1 | Met | Yes | 2006Q2 to 2025Q4 | Expanded outcome; Tier A plus Tier B |
| Expanded Tier A+B | Core | Alemtuzumab | Tier A+B | Contemporaneous active DMTs | 39 | 3045 | 465 | 218353 | 3084 | 218818 | 3.39 (2.41 to 4.78) | 2.90 × 10⁻¹² | 1.45 × 10⁻¹¹ | 95% CI above 1 | Met | Yes | 2014Q4 to 2025Q4 | Expanded outcome; Tier A plus Tier B |
| Expanded Tier A+B | Core | Cladribine | Tier A+B | Contemporaneous active DMTs | 6 | 3071 | 340 | 123364 | 3077 | 123704 | 0.61 (0.28 to 1.32) | 0.211 | 0.264 | 95% CI includes 1 | Met | Yes | 2019Q2 to 2025Q4 | Expanded outcome; Tier A plus Tier B |
| Exploratory Tier A+B+C | Exploratory | Anti-CD20 core | Tier A+B+C | Contemporaneous active DMTs | 245 | 42549 | 443 | 127172 | 42794 | 127615 | 0.97 (0.81 to 1.17) | 0.768 | 0.768 | 95% CI includes 1 | Met | Yes | 2017Q2 to 2025Q4 | Wide outcome including uncertain Tier C |
| Exploratory Tier A+B+C | Exploratory | S1P modulators | Tier A+B+C | Contemporaneous active DMTs | 221 | 60229 | 732 | 266803 | 60450 | 267535 | 1.32 (1.13 to 1.55) | 6.47 × 10⁻⁴ | 0.001 | 95% CI above 1 | Met | Yes | 2010Q4 to 2025Q4 | Wide outcome including uncertain Tier C |
| Exploratory Tier A+B+C | Exploratory | Natalizumab | Tier A+B+C | Contemporaneous active DMTs | 313 | 131786 | 683 | 211277 | 132099 | 211960 | 1.39 (1.19 to 1.63) | 5.02 × 10⁻⁵ | 1.25 × 10⁻⁴ | 95% CI above 1 | Met | Yes | 2006Q2 to 2025Q4 | Wide outcome including uncertain Tier C |
| Exploratory Tier A+B+C | Exploratory | Alemtuzumab | Tier A+B+C | Contemporaneous active DMTs | 51 | 3033 | 772 | 218046 | 3084 | 218818 | 2.65 (1.97 to 3.56) | 1.37 × 10⁻¹⁰ | 6.86 × 10⁻¹⁰ | 95% CI above 1 | Met | Yes | 2014Q4 to 2025Q4 | Wide outcome including uncertain Tier C |
| Exploratory Tier A+B+C | Exploratory | Cladribine | Tier A+B+C | Contemporaneous active DMTs | 12 | 3065 | 553 | 123151 | 3077 | 123704 | 0.72 (0.41 to 1.27) | 0.256 | 0.320 | 95% CI includes 1 | Met | Yes | 2019Q2 to 2025Q4 | Wide outcome including uncertain Tier C |
| PML/JCV reports removed from denominator | Core | Anti-CD20 core | Tier A | Contemporaneous active DMTs | 122 | 42628 | 253 | 126583 | 42750 | 126836 | 0.80 (0.62 to 1.03) | 0.086 | 0.107 | 95% CI includes 1 | Met | Yes | 2017Q2 to 2025Q4 | All PML/JCV reports removed from denominator |
| PML/JCV reports removed from denominator | Core | S1P modulators | Tier A | Contemporaneous active DMTs | 152 | 60083 | 367 | 265594 | 60235 | 265961 | 1.75 (1.43 to 2.13) | 4.29 × 10⁻⁸ | 1.07 × 10⁻⁷ | 95% CI above 1 | Met | Yes | 2010Q4 to 2025Q4 | All PML/JCV reports removed from denominator |
| PML/JCV reports removed from denominator | Core | Natalizumab | Tier A | Contemporaneous active DMTs | 159 | 130465 | 382 | 211223 | 130624 | 211605 | 1.41 (1.14 to 1.75) | 0.002 | 0.003 | 95% CI above 1 | Met | Yes | 2006Q2 to 2025Q4 | All PML/JCV reports removed from denominator |
| PML/JCV reports removed from denominator | Core | Alemtuzumab | Tier A | Contemporaneous active DMTs | 35 | 3046 | 418 | 216918 | 3081 | 217336 | 3.19 (2.22 to 4.58) | 3.12 × 10⁻¹⁰ | 1.56 × 10⁻⁹ | 95% CI above 1 | Met | Yes | 2014Q4 to 2025Q4 | All PML/JCV reports removed from denominator |
| PML/JCV reports removed from denominator | Core | Cladribine | Tier A | Contemporaneous active DMTs | 5 | 3070 | 303 | 122818 | 3075 | 123121 | 0.55 (0.24 to 1.28) | 0.169 | 0.169 | 95% CI includes 1 | Met | Yes | 2019Q2 to 2025Q4 | All PML/JCV reports removed from denominator |
| Infection-report denominator | Core | Anti-CD20 core | Tier A | Contemporaneous active DMTs | 122 | 12574 | 253 | 7337 | 12696 | 7590 | 0.26 (0.20 to 0.34) | 2.68 × 10⁻²³ | 1.34 × 10⁻²² | 95% CI below 1 | Met | Yes | 2017Q2 to 2025Q4 | Tier A CNS infection versus other non-PML infection-related reports |
| Infection-report denominator | Core | S1P modulators | Tier A | Contemporaneous active DMTs | 152 | 3675 | 367 | 23762 | 3827 | 24129 | 2.52 (2.05 to 3.10) | 2.26 × 10⁻¹⁸ | 5.66 × 10⁻¹⁸ | 95% CI above 1 | Met | Yes | 2010Q4 to 2025Q4 | Tier A CNS infection versus other non-PML infection-related reports |
| Infection-report denominator | Core | Natalizumab | Tier A | Contemporaneous active DMTs | 159 | 7484 | 382 | 21022 | 7643 | 21404 | 1.58 (1.24 to 2.01) | 1.89 × 10⁻⁴ | 3.15 × 10⁻⁴ | 95% CI above 1 | Met | Yes | 2006Q2 to 2025Q4 | Tier A CNS infection versus other non-PML infection-related reports |
| Infection-report denominator | Core | Alemtuzumab | Tier A | Contemporaneous active DMTs | 35 | 610 | 418 | 21565 | 645 | 21983 | 1.46 (1.00 to 2.14) | 0.048 | 0.060 | 95% CI above 1 | Met | Yes | 2014Q4 to 2025Q4 | Tier A CNS infection versus other non-PML infection-related reports |
| Infection-report denominator | Core | Cladribine | Tier A | Contemporaneous active DMTs | 5 | 245 | 303 | 17476 | 250 | 17779 | 0.79 (0.34 to 1.87) | 0.596 | 0.596 | 95% CI includes 1 | Met | Yes | 2019Q2 to 2025Q4 | Tier A CNS infection versus other non-PML infection-related reports |
| Interferon-beta/glatiramer acetate comparator | Secondary | Anti-CD20 core | Tier A | Interferon-beta/glatiramer acetate | 122 | 42672 | 19 | 39557 | 42794 | 39576 | 6.25 (3.59 to 10.87) | 8.88 × 10⁻¹¹ | 8.88 × 10⁻¹¹ | 95% CI above 1 | Met | Yes | 2017Q2 to 2025Q4 | Low-immunosuppression comparator |
| Interferon-beta/glatiramer acetate comparator | Secondary | S1P modulators | Tier A | Interferon-beta/glatiramer acetate | 152 | 60298 | 66 | 109411 | 60450 | 109477 | 3.94 (2.88 to 5.39) | 8.66 × 10⁻¹⁸ | 1.73 × 10⁻¹⁷ | 95% CI above 1 | Met | Yes | 2010Q4 to 2025Q4 | Low-immunosuppression comparator |
| Interferon-beta/glatiramer acetate comparator | Secondary | Natalizumab | Tier A | Interferon-beta/glatiramer acetate | 159 | 131940 | 105 | 136566 | 132099 | 136671 | 2.39 (1.84 to 3.09) | 4.89 × 10⁻¹¹ | 6.52 × 10⁻¹¹ | 95% CI above 1 | Met | Yes | 2006Q2 to 2025Q4 | Low-immunosuppression comparator |
| Interferon-beta/glatiramer acetate comparator | Secondary | Alemtuzumab | Tier A | Interferon-beta/glatiramer acetate | 35 | 3049 | 35 | 67733 | 3084 | 67768 | 11.52 (6.96 to 19.08) | 2.10 × 10⁻²¹ | 8.41 × 10⁻²¹ | 95% CI above 1 | Met | Yes | 2014Q4 to 2025Q4 | Low-immunosuppression comparator |
| Interferon-beta/glatiramer acetate comparator | Secondary | Cladribine | Tier A | Interferon-beta/glatiramer acetate | 5 | 3072 | 12 | 22309 | 3077 | 22321 | NE | NE | NE | Not estimable | Not met | No/not fitted | 2019Q2 to 2025Q4 | Low-immunosuppression comparator |
| All other MS-DMT comparator | Secondary | Anti-CD20 core | Tier A | All other mapped MS DMTs | 122 | 42672 | 282 | 169969 | 42794 | 170251 | 1.00 (0.78 to 1.29) | 0.994 | 0.994 | 95% CI includes 1 | Met | Yes | 2017Q2 to 2025Q4 | All other mapped MS-DMT comparator |
| All other MS-DMT comparator | Secondary | S1P modulators | Tier A | All other mapped MS DMTs | 152 | 60298 | 444 | 379750 | 60450 | 380194 | 2.13 (1.75 to 2.58) | 2.00 × 10⁻¹⁴ | 1.00 × 10⁻¹³ | 95% CI above 1 | Met | Yes | 2010Q4 to 2025Q4 | All other mapped MS-DMT comparator |
| All other MS-DMT comparator | Secondary | Natalizumab | Tier A | All other mapped MS DMTs | 159 | 131940 | 498 | 351334 | 132099 | 351832 | 1.50 (1.23 to 1.82) | 4.17 × 10⁻⁵ | 6.94 × 10⁻⁵ | 95% CI above 1 | Met | Yes | 2006Q2 to 2025Q4 | All other mapped MS-DMT comparator |
| All other MS-DMT comparator | Secondary | Alemtuzumab | Tier A | All other mapped MS DMTs | 35 | 3049 | 464 | 289292 | 3084 | 289756 | 3.96 (2.77 to 5.66) | 4.17 × 10⁻¹⁴ | 1.04 × 10⁻¹³ | 95% CI above 1 | Met | Yes | 2014Q4 to 2025Q4 | All other mapped MS-DMT comparator |
| All other MS-DMT comparator | Secondary | Cladribine | Tier A | All other mapped MS DMTs | 5 | 3072 | 324 | 148472 | 3077 | 148796 | 0.66 (0.28 to 1.52) | 0.327 | 0.409 | 95% CI includes 1 | Met | Yes | 2019Q2 to 2025Q4 | All other mapped MS-DMT comparator |
| Healthcare-professional reports only | Core | Anti-CD20 core | Tier A | Contemporaneous active DMTs | 92 | 14193 | 188 | 38733 | 14285 | 38921 | 0.98 (0.75 to 1.29) | 0.909 | 0.909 | 95% CI includes 1 | Met | Yes | 2017Q2 to 2025Q4 | Healthcare-professional reporters |
| Healthcare-professional reports only | Core | S1P modulators | Tier A | Contemporaneous active DMTs | 112 | 19295 | 237 | 57431 | 19407 | 57668 | 1.33 (1.06 to 1.67) | 0.013 | 0.033 | 95% CI above 1 | Met | Yes | 2010Q4 to 2025Q4 | Healthcare-professional reporters |
| Healthcare-professional reports only | Core | Natalizumab | Tier A | Contemporaneous active DMTs | 87 | 20160 | 270 | 58874 | 20247 | 59144 | 1.27 (0.97 to 1.66) | 0.078 | 0.098 | 95% CI includes 1 | Met | Yes | 2006Q2 to 2025Q4 | Healthcare-professional reporters |
| Healthcare-professional reports only | Core | Alemtuzumab | Tier A | Contemporaneous active DMTs | 21 | 1333 | 299 | 62867 | 1354 | 63166 | 2.51 (1.60 to 3.95) | 6.58 × 10⁻⁵ | 3.29 × 10⁻⁴ | 95% CI above 1 | Met | Yes | 2014Q4 to 2025Q4 | Healthcare-professional reporters |
| Healthcare-professional reports only | Core | Cladribine | Tier A | Contemporaneous active DMTs | 1 | 1061 | 232 | 42856 | 1062 | 43088 | 0.23 (0.05 to 1.14) | 0.072 | 0.098 | 95% CI includes 1 | Met | Yes | 2019Q2 to 2025Q4 | Healthcare-professional reporters |
| Serious reports only | Core | Anti-CD20 core | Tier A | Contemporaneous active DMTs | 120 | 20057 | 252 | 55900 | 20177 | 56152 | 0.97 (0.76 to 1.25) | 0.829 | 0.829 | 95% CI includes 1 | Met | Yes | 2017Q2 to 2025Q4 | Reports with a serious outcome code |
| Serious reports only | Core | S1P modulators | Tier A | Contemporaneous active DMTs | 151 | 29257 | 352 | 88120 | 29408 | 88472 | 1.48 (1.21 to 1.81) | 1.44 × 10⁻⁴ | 3.61 × 10⁻⁴ | 95% CI above 1 | Met | Yes | 2010Q4 to 2025Q4 | Reports with a serious outcome code |
| Serious reports only | Core | Natalizumab | Tier A | Contemporaneous active DMTs | 147 | 35103 | 377 | 88372 | 35250 | 88749 | 1.30 (1.05 to 1.61) | 0.017 | 0.028 | 95% CI above 1 | Met | Yes | 2006Q2 to 2025Q4 | Reports with a serious outcome code |
| Serious reports only | Core | Alemtuzumab | Tier A | Contemporaneous active DMTs | 35 | 2583 | 414 | 94497 | 2618 | 94911 | 2.51 (1.75 to 3.59) | 5.18 × 10⁻⁷ | 2.59 × 10⁻⁶ | 95% CI above 1 | Met | Yes | 2014Q4 to 2025Q4 | Reports with a serious outcome code |
| Serious reports only | Core | Cladribine | Tier A | Contemporaneous active DMTs | 5 | 1768 | 300 | 57249 | 1773 | 57549 | 0.52 (0.22 to 1.20) | 0.123 | 0.154 | 95% CI includes 1 | Met | Yes | 2019Q2 to 2025Q4 | Reports with a serious outcome code |
| Literature/study reports excluded | Core | Anti-CD20 core | Tier A | Contemporaneous active DMTs | 122 | 42672 | 253 | 127362 | 42794 | 127615 | 0.82 (0.63 to 1.05) | 0.115 | 0.144 | 95% CI includes 1 | Met | Yes | 2017Q2 to 2025Q4 | Literature/study reports excluded |
| Literature/study reports excluded | Core | S1P modulators | Tier A | Contemporaneous active DMTs | 152 | 60279 | 366 | 267043 | 60431 | 267409 | 1.77 (1.45 to 2.16) | 1.97 × 10⁻⁸ | 4.93 × 10⁻⁸ | 95% CI above 1 | Met | Yes | 2010Q4 to 2025Q4 | Literature/study reports excluded |
| Literature/study reports excluded | Core | Natalizumab | Tier A | Contemporaneous active DMTs | 157 | 131211 | 381 | 211341 | 131368 | 211722 | 1.32 (1.06 to 1.64) | 0.012 | 0.021 | 95% CI above 1 | Met | Yes | 2006Q2 to 2025Q4 | Literature/study reports excluded |
| Literature/study reports excluded | Core | Alemtuzumab | Tier A | Contemporaneous active DMTs | 35 | 3046 | 418 | 218316 | 3081 | 218734 | 3.28 (2.29 to 4.71) | 1.11 × 10⁻¹⁰ | 5.53 × 10⁻¹⁰ | 95% CI above 1 | Met | Yes | 2014Q4 to 2025Q4 | Literature/study reports excluded |
| Literature/study reports excluded | Core | Cladribine | Tier A | Contemporaneous active DMTs | 5 | 3072 | 303 | 123401 | 3077 | 123704 | 0.56 (0.24 to 1.30) | 0.177 | 0.177 | 95% CI includes 1 | Met | Yes | 2019Q2 to 2025Q4 | Literature/study reports excluded |
| Steroid PS/SS reports excluded | Core | Anti-CD20 core | Tier A | Contemporaneous active DMTs | 122 | 42672 | 253 | 127362 | 42794 | 127615 | 0.82 (0.63 to 1.05) | 0.115 | 0.144 | 95% CI includes 1 | Met | Yes | 2017Q2 to 2025Q4 | Steroid PS/SS reports excluded |
| Steroid PS/SS reports excluded | Core | S1P modulators | Tier A | Contemporaneous active DMTs | 152 | 60298 | 367 | 267168 | 60450 | 267535 | 1.77 (1.45 to 2.16) | 2.13 × 10⁻⁸ | 5.32 × 10⁻⁸ | 95% CI above 1 | Met | Yes | 2010Q4 to 2025Q4 | Steroid PS/SS reports excluded |
| Steroid PS/SS reports excluded | Core | Natalizumab | Tier A | Contemporaneous active DMTs | 159 | 131940 | 382 | 211578 | 132099 | 211960 | 1.32 (1.06 to 1.64) | 0.012 | 0.020 | 95% CI above 1 | Met | Yes | 2006Q2 to 2025Q4 | Steroid PS/SS reports excluded |
| Steroid PS/SS reports excluded | Core | Alemtuzumab | Tier A | Contemporaneous active DMTs | 35 | 3049 | 418 | 218400 | 3084 | 218818 | 3.28 (2.29 to 4.71) | 1.12 × 10⁻¹⁰ | 5.60 × 10⁻¹⁰ | 95% CI above 1 | Met | Yes | 2014Q4 to 2025Q4 | Steroid PS/SS reports excluded |
| Steroid PS/SS reports excluded | Core | Cladribine | Tier A | Contemporaneous active DMTs | 5 | 3072 | 303 | 123401 | 3077 | 123704 | 0.56 (0.24 to 1.30) | 0.177 | 0.177 | 95% CI includes 1 | Met | Yes | 2019Q2 to 2025Q4 | Steroid PS/SS reports excluded |
| Any co-reported steroid excluded | Core | Anti-CD20 core | Tier A | Contemporaneous active DMTs | 116 | 41062 | 238 | 125189 | 41178 | 125427 | 0.81 (0.62 to 1.04) | 0.104 | 0.129 | 95% CI includes 1 | Met | Yes | 2017Q2 to 2025Q4 | Any co-reported steroid excluded |
| Any co-reported steroid excluded | Core | S1P modulators | Tier A | Contemporaneous active DMTs | 146 | 59297 | 340 | 263064 | 59443 | 263404 | 1.86 (1.51 to 2.28) | 2.85 × 10⁻⁹ | 1.42 × 10⁻⁸ | 95% CI above 1 | Met | Yes | 2010Q4 to 2025Q4 | Any co-reported steroid excluded |
| Any co-reported steroid excluded | Core | Natalizumab | Tier A | Contemporaneous active DMTs | 152 | 131290 | 356 | 207041 | 131442 | 207397 | 1.32 (1.06 to 1.65) | 0.015 | 0.024 | 95% CI above 1 | Met | Yes | 2006Q2 to 2025Q4 | Any co-reported steroid excluded |
| Any co-reported steroid excluded | Core | Alemtuzumab | Tier A | Contemporaneous active DMTs | 22 | 2569 | 398 | 214189 | 2591 | 214587 | 2.86 (1.85 to 4.44) | 2.57 × 10⁻⁶ | 6.43 × 10⁻⁶ | 95% CI above 1 | Met | Yes | 2014Q4 to 2025Q4 | Any co-reported steroid excluded |
| Any co-reported steroid excluded | Core | Cladribine | Tier A | Contemporaneous active DMTs | 5 | 3045 | 293 | 120560 | 3050 | 120853 | 0.57 (0.25 to 1.33) | 0.196 | 0.196 | 95% CI includes 1 | Met | Yes | 2019Q2 to 2025Q4 | Any co-reported steroid excluded |
| Strong immunosuppressant co-reports excluded | Core | Anti-CD20 core | Tier A | Contemporaneous active DMTs | 122 | 42568 | 253 | 127145 | 42690 | 127398 | 0.82 (0.63 to 1.05) | 0.116 | 0.146 | 95% CI includes 1 | Met | Yes | 2017Q2 to 2025Q4 | Strong immunosuppressant/antineoplastic co-reports excluded |
| Strong immunosuppressant co-reports excluded | Core | S1P modulators | Tier A | Contemporaneous active DMTs | 151 | 60200 | 367 | 266791 | 60351 | 267158 | 1.77 (1.45 to 2.16) | 2.21 × 10⁻⁸ | 5.52 × 10⁻⁸ | 95% CI above 1 | Met | Yes | 2010Q4 to 2025Q4 | Strong immunosuppressant/antineoplastic co-reports excluded |
| Strong immunosuppressant co-reports excluded | Core | Natalizumab | Tier A | Contemporaneous active DMTs | 159 | 131859 | 381 | 211160 | 132018 | 211541 | 1.32 (1.06 to 1.64) | 0.012 | 0.020 | 95% CI above 1 | Met | Yes | 2006Q2 to 2025Q4 | Strong immunosuppressant/antineoplastic co-reports excluded |
| Strong immunosuppressant co-reports excluded | Core | Alemtuzumab | Tier A | Contemporaneous active DMTs | 35 | 3038 | 417 | 217982 | 3073 | 218399 | 3.28 (2.28 to 4.70) | 1.16 × 10⁻¹⁰ | 5.81 × 10⁻¹⁰ | 95% CI above 1 | Met | Yes | 2014Q4 to 2025Q4 | Strong immunosuppressant/antineoplastic co-reports excluded |
| Strong immunosuppressant co-reports excluded | Core | Cladribine | Tier A | Contemporaneous active DMTs | 5 | 3070 | 303 | 123157 | 3075 | 123460 | 0.56 (0.24 to 1.30) | 0.176 | 0.176 | 95% CI includes 1 | Met | Yes | 2019Q2 to 2025Q4 | Strong immunosuppressant/antineoplastic co-reports excluded |
| Six-month carryover exclusion | Core | Anti-CD20 core | Tier A | Contemporaneous active DMTs | 122 | 42672 | 253 | 127362 | 42794 | 127615 | 0.82 (0.63 to 1.05) | 0.115 | 0.144 | 95% CI includes 1 | Met | Yes | 2017Q2 to 2025Q4 | Reports within 183 days of another high-efficacy DMT end date excluded |
| Six-month carryover exclusion | Core | S1P modulators | Tier A | Contemporaneous active DMTs | 152 | 60298 | 367 | 267166 | 60450 | 267533 | 1.77 (1.45 to 2.16) | 2.13 × 10⁻⁸ | 5.32 × 10⁻⁸ | 95% CI above 1 | Met | Yes | 2010Q4 to 2025Q4 | Reports within 183 days of another high-efficacy DMT end date excluded |
| Six-month carryover exclusion | Core | Natalizumab | Tier A | Contemporaneous active DMTs | 159 | 131938 | 382 | 211578 | 132097 | 211960 | 1.32 (1.06 to 1.64) | 0.012 | 0.020 | 95% CI above 1 | Met | Yes | 2006Q2 to 2025Q4 | Reports within 183 days of another high-efficacy DMT end date excluded |
| Six-month carryover exclusion | Core | Alemtuzumab | Tier A | Contemporaneous active DMTs | 35 | 3049 | 418 | 218400 | 3084 | 218818 | 3.28 (2.29 to 4.71) | 1.12 × 10⁻¹⁰ | 5.60 × 10⁻¹⁰ | 95% CI above 1 | Met | Yes | 2014Q4 to 2025Q4 | Reports within 183 days of another high-efficacy DMT end date excluded |
| Six-month carryover exclusion | Core | Cladribine | Tier A | Contemporaneous active DMTs | 5 | 3072 | 303 | 123401 | 3077 | 123704 | 0.56 (0.24 to 1.30) | 0.177 | 0.177 | 95% CI includes 1 | Met | Yes | 2019Q2 to 2025Q4 | Reports within 183 days of another high-efficacy DMT end date excluded |
| Six-/12-month carryover exclusion | Core | Anti-CD20 core | Tier A | Contemporaneous active DMTs | 122 | 42672 | 253 | 127362 | 42794 | 127615 | 0.82 (0.63 to 1.05) | 0.115 | 0.144 | 95% CI includes 1 | Met | Yes | 2017Q2 to 2025Q4 | Six-month window for any high-efficacy DMT and 12-month window for persistent mechanisms |
| Six-/12-month carryover exclusion | Core | S1P modulators | Tier A | Contemporaneous active DMTs | 152 | 60298 | 367 | 267166 | 60450 | 267533 | 1.77 (1.45 to 2.16) | 2.13 × 10⁻⁸ | 5.32 × 10⁻⁸ | 95% CI above 1 | Met | Yes | 2010Q4 to 2025Q4 | Six-month window for any high-efficacy DMT and 12-month window for persistent mechanisms |
| Six-/12-month carryover exclusion | Core | Natalizumab | Tier A | Contemporaneous active DMTs | 159 | 131938 | 382 | 211578 | 132097 | 211960 | 1.32 (1.06 to 1.64) | 0.012 | 0.020 | 95% CI above 1 | Met | Yes | 2006Q2 to 2025Q4 | Six-month window for any high-efficacy DMT and 12-month window for persistent mechanisms |
| Six-/12-month carryover exclusion | Core | Alemtuzumab | Tier A | Contemporaneous active DMTs | 35 | 3049 | 418 | 218400 | 3084 | 218818 | 3.28 (2.29 to 4.71) | 1.12 × 10⁻¹⁰ | 5.60 × 10⁻¹⁰ | 95% CI above 1 | Met | Yes | 2014Q4 to 2025Q4 | Six-month window for any high-efficacy DMT and 12-month window for persistent mechanisms |
| Six-/12-month carryover exclusion | Core | Cladribine | Tier A | Contemporaneous active DMTs | 5 | 3072 | 303 | 123401 | 3077 | 123704 | 0.56 (0.24 to 1.30) | 0.177 | 0.177 | 95% CI includes 1 | Met | Yes | 2019Q2 to 2025Q4 | Six-month window for any high-efficacy DMT and 12-month window for persistent mechanisms |
| Pandemic years 2020–2021 excluded | Core | Anti-CD20 core | Tier A | Contemporaneous active DMTs | 99 | 34785 | 197 | 94156 | 34884 | 94353 | 0.80 (0.60 to 1.05) | 0.112 | 0.112 | 95% CI includes 1 | Met | Yes | 2017Q2 to 2025Q4 | Pandemic calendar years excluded |
| Pandemic years 2020–2021 excluded | Core | S1P modulators | Tier A | Contemporaneous active DMTs | 121 | 49580 | 319 | 236793 | 49701 | 237112 | 1.68 (1.35 to 2.10) | 3.78 × 10⁻⁶ | 9.45 × 10⁻⁶ | 95% CI above 1 | Met | Yes | 2010Q4 to 2025Q4 | Pandemic calendar years excluded |
| Pandemic years 2020–2021 excluded | Core | Natalizumab | Tier A | Contemporaneous active DMTs | 150 | 126589 | 312 | 175836 | 126739 | 176148 | 1.39 (1.10 to 1.75) | 0.005 | 0.008 | 95% CI above 1 | Met | Yes | 2006Q2 to 2025Q4 | Pandemic calendar years excluded |
| Pandemic years 2020–2021 excluded | Core | Alemtuzumab | Tier A | Contemporaneous active DMTs | 33 | 2521 | 341 | 177835 | 2554 | 178176 | 3.90 (2.67 to 5.69) | 1.94 × 10⁻¹² | 9.70 × 10⁻¹² | 95% CI above 1 | Met | Yes | 2014Q4 to 2025Q4 | Pandemic calendar years excluded |
| Pandemic years 2020–2021 excluded | Core | Cladribine | Tier A | Contemporaneous active DMTs | 3 | 2336 | 226 | 83044 | 2339 | 83270 | 0.42 (0.15 to 1.21) | 0.109 | 0.112 | 95% CI includes 1 | Met | Yes | 2019Q2 to 2025Q4 | Pandemic calendar years excluded |
| Relapsing/CIS/active-SPMS only | Core | Anti-CD20 core | Tier A | Contemporaneous active DMTs | 54 | 18275 | 73 | 10364 | 18329 | 10437 | 0.43 (0.30 to 0.64) | 1.91 × 10⁻⁵ | 9.56 × 10⁻⁵ | 95% CI below 1 | Met | Yes | 2017Q2 to 2025Q4 | Strict relapsing/CIS/active-SPMS indications |
| Relapsing/CIS/active-SPMS only | Core | S1P modulators | Tier A | Contemporaneous active DMTs | 58 | 10015 | 103 | 26725 | 10073 | 26828 | 1.69 (1.21 to 2.36) | 0.002 | 0.005 | 95% CI above 1 | Met | Yes | 2010Q4 to 2025Q4 | Strict relapsing/CIS/active-SPMS indications |
| Relapsing/CIS/active-SPMS only | Core | Natalizumab | Tier A | Contemporaneous active DMTs | 23 | 3134 | 139 | 33925 | 3157 | 34064 | 1.50 (0.93 to 2.42) | 0.095 | 0.158 | 95% CI includes 1 | Met | Yes | 2006Q2 to 2025Q4 | Strict relapsing/CIS/active-SPMS indications |
| Relapsing/CIS/active-SPMS only | Core | Alemtuzumab | Tier A | Contemporaneous active DMTs | 9 | 669 | 144 | 32003 | 678 | 32147 | 1.67 (0.85 to 3.31) | 0.138 | 0.173 | 95% CI includes 1 | Met | Yes | 2014Q4 to 2025Q4 | Strict relapsing/CIS/active-SPMS indications |
| Relapsing/CIS/active-SPMS only | Core | Cladribine | Tier A | Contemporaneous active DMTs | 0 | 223 | 109 | 24799 | 223 | 24908 | 0.37 (0.03 to 5.42) | 0.467 | 0.467 | 95% CI includes 1 | Met | Yes | 2019Q2 to 2025Q4 | Strict relapsing/CIS/active-SPMS indications |
| PPMS indications excluded | Core | Anti-CD20 core | Tier A | Contemporaneous active DMTs | 115 | 39036 | 253 | 127294 | 39151 | 127547 | 0.81 (0.63 to 1.04) | 0.095 | 0.119 | 95% CI includes 1 | Met | Yes | 2017Q2 to 2025Q4 | Explicit PPMS indications excluded |
| PPMS indications excluded | Core | S1P modulators | Tier A | Contemporaneous active DMTs | 152 | 60221 | 360 | 263488 | 60373 | 263848 | 1.77 (1.45 to 2.17) | 1.85 × 10⁻⁸ | 4.61 × 10⁻⁸ | 95% CI above 1 | Met | Yes | 2010Q4 to 2025Q4 | Explicit PPMS indications excluded |
| PPMS indications excluded | Core | Natalizumab | Tier A | Contemporaneous active DMTs | 159 | 131920 | 375 | 207839 | 132079 | 208214 | 1.32 (1.06 to 1.64) | 0.012 | 0.019 | 95% CI above 1 | Met | Yes | 2006Q2 to 2025Q4 | Explicit PPMS indications excluded |
| PPMS indications excluded | Core | Alemtuzumab | Tier A | Contemporaneous active DMTs | 35 | 3047 | 411 | 214656 | 3082 | 215067 | 3.28 (2.28 to 4.71) | 1.18 × 10⁻¹⁰ | 5.92 × 10⁻¹⁰ | 95% CI above 1 | Met | Yes | 2014Q4 to 2025Q4 | Explicit PPMS indications excluded |
| PPMS indications excluded | Core | Cladribine | Tier A | Contemporaneous active DMTs | 5 | 3071 | 296 | 120171 | 3076 | 120467 | 0.56 (0.24 to 1.29) | 0.173 | 0.173 | 95% CI includes 1 | Met | Yes | 2019Q2 to 2025Q4 | Explicit PPMS indications excluded |
| Adult complete-case analysis | Core | Anti-CD20 core | Tier A | Contemporaneous active DMTs | 80 | 21917 | 191 | 56257 | 21997 | 56448 | 0.68 (0.50 to 0.92) | 0.013 | 0.022 | 95% CI below 1 | Met | Yes | 2017Q2 to 2025Q4 | Adult complete cases for age, sex, and reporter occupation |
| Adult complete-case analysis | Core | S1P modulators | Tier A | Contemporaneous active DMTs | 125 | 33906 | 252 | 146568 | 34031 | 146820 | 1.96 (1.56 to 2.45) | 4.05 × 10⁻⁹ | 2.03 × 10⁻⁸ | 95% CI above 1 | Met | Yes | 2010Q4 to 2025Q4 | Adult complete cases for age, sex, and reporter occupation |
| Adult complete-case analysis | Core | Natalizumab | Tier A | Contemporaneous active DMTs | 116 | 84558 | 277 | 109424 | 84674 | 109701 | 1.28 (0.99 to 1.66) | 0.057 | 0.071 | 95% CI includes 1 | Met | Yes | 2006Q2 to 2025Q4 | Adult complete cases for age, sex, and reporter occupation |
| Adult complete-case analysis | Core | Alemtuzumab | Tier A | Contemporaneous active DMTs | 29 | 2367 | 296 | 103501 | 2396 | 103797 | 3.13 (2.10 to 4.65) | 1.95 × 10⁻⁸ | 4.88 × 10⁻⁸ | 95% CI above 1 | Met | Yes | 2014Q4 to 2025Q4 | Adult complete cases for age, sex, and reporter occupation |
| Adult complete-case analysis | Core | Cladribine | Tier A | Contemporaneous active DMTs | 4 | 1436 | 212 | 57246 | 1440 | 57458 | 0.77 (0.30 to 1.94) | 0.578 | 0.578 | 95% CI includes 1 | Met | Yes | 2019Q2 to 2025Q4 | Adult complete cases for age, sex, and reporter occupation |
| Target DMT was the only PS drug | Core | Anti-CD20 core | Tier A | Contemporaneous active DMTs | 122 | 42672 | 253 | 127362 | 42794 | 127615 | 0.82 (0.63 to 1.05) | 0.115 | 0.144 | 95% CI includes 1 | Met | Yes | 2017Q2 to 2025Q4 | Target DMT is the only PS drug of any type |
| Target DMT was the only PS drug | Core | S1P modulators | Tier A | Contemporaneous active DMTs | 152 | 60298 | 367 | 267168 | 60450 | 267535 | 1.77 (1.45 to 2.16) | 2.13 × 10⁻⁸ | 5.32 × 10⁻⁸ | 95% CI above 1 | Met | Yes | 2010Q4 to 2025Q4 | Target DMT is the only PS drug of any type |
| Target DMT was the only PS drug | Core | Natalizumab | Tier A | Contemporaneous active DMTs | 159 | 131940 | 382 | 211578 | 132099 | 211960 | 1.32 (1.06 to 1.64) | 0.012 | 0.020 | 95% CI above 1 | Met | Yes | 2006Q2 to 2025Q4 | Target DMT is the only PS drug of any type |
| Target DMT was the only PS drug | Core | Alemtuzumab | Tier A | Contemporaneous active DMTs | 35 | 3049 | 418 | 218400 | 3084 | 218818 | 3.28 (2.29 to 4.71) | 1.12 × 10⁻¹⁰ | 5.60 × 10⁻¹⁰ | 95% CI above 1 | Met | Yes | 2014Q4 to 2025Q4 | Target DMT is the only PS drug of any type |
| Target DMT was the only PS drug | Core | Cladribine | Tier A | Contemporaneous active DMTs | 5 | 3072 | 303 | 123401 | 3077 | 123704 | 0.56 (0.24 to 1.30) | 0.177 | 0.177 | 95% CI includes 1 | Met | Yes | 2019Q2 to 2025Q4 | Target DMT is the only PS drug of any type |
| First two post-launch years excluded | Core | Anti-CD20 core | Tier A | Contemporaneous active DMTs | 114 | 39883 | 194 | 86590 | 39997 | 86784 | 0.78 (0.60 to 1.01) | 0.060 | 0.075 | 95% CI includes 1 | Met | Yes | 2019Q2 to 2025Q4 | First eight post-launch quarters excluded |
| First two post-launch years excluded | Core | S1P modulators | Tier A | Contemporaneous active DMTs | 149 | 56884 | 355 | 229987 | 57033 | 230342 | 1.77 (1.45 to 2.17) | 2.91 × 10⁻⁸ | 1.46 × 10⁻⁷ | 95% CI above 1 | Met | Yes | 2012Q4 to 2025Q4 | First eight post-launch quarters excluded |
| First two post-launch years excluded | Core | Natalizumab | Tier A | Contemporaneous active DMTs | 157 | 131202 | 382 | 211420 | 131359 | 211802 | 1.32 (1.06 to 1.63) | 0.013 | 0.022 | 95% CI above 1 | Met | Yes | 2008Q2 to 2025Q4 | First eight post-launch quarters excluded |
| First two post-launch years excluded | Core | Alemtuzumab | Tier A | Contemporaneous active DMTs | 26 | 2546 | 370 | 178580 | 2572 | 178950 | 2.79 (1.85 to 4.22) | 1.04 × 10⁻⁶ | 2.60 × 10⁻⁶ | 95% CI above 1 | Met | Yes | 2016Q4 to 2025Q4 | First eight post-launch quarters excluded |
| First two post-launch years excluded | Core | Cladribine | Tier A | Contemporaneous active DMTs | 4 | 2372 | 231 | 82415 | 2376 | 82646 | 0.53 (0.21 to 1.34) | 0.181 | 0.181 | 95% CI includes 1 | Met | Yes | 2021Q2 to 2025Q4 | First eight post-launch quarters excluded |
| PS+SS exposure expansion | Core | Anti-CD20 core | Tier A | Active DMTs; PS/SS target definition | 177 | 61923 | 398 | 186254 | 62100 | 186652 | 0.77 (0.62 to 0.95) | 0.015 | 0.018 | 95% CI below 1 | Met | Yes | 2017Q2 to 2025Q4 | Any eligible target PS/SS report classified as target; reports containing target plus another DMT remain target in this role-misclassification sensitivity |
| PS+SS exposure expansion | Core | S1P modulators | Tier A | Active DMTs; PS/SS target definition | 199 | 74451 | 571 | 368987 | 74650 | 369558 | 1.65 (1.39 to 1.96) | 9.78 × 10⁻⁹ | 4.89 × 10⁻⁸ | 95% CI above 1 | Met | Yes | 2010Q4 to 2025Q4 | Any eligible target PS/SS report classified as target; reports containing target plus another DMT remain target in this role-misclassification sensitivity |
| PS+SS exposure expansion | Core | Natalizumab | Tier A | Active DMTs; PS/SS target definition | 242 | 165283 | 553 | 296092 | 165525 | 296645 | 1.62 (1.36 to 1.93) | 4.83 × 10⁻⁸ | 1.21 × 10⁻⁷ | 95% CI above 1 | Met | Yes | 2006Q2 to 2025Q4 | Any eligible target PS/SS report classified as target; reports containing target plus another DMT remain target in this role-misclassification sensitivity |
| PS+SS exposure expansion | Core | Alemtuzumab | Tier A | Active DMTs; PS/SS target definition | 49 | 6231 | 635 | 307630 | 6280 | 308265 | 1.92 (1.42 to 2.60) | 2.22 × 10⁻⁵ | 3.70 × 10⁻⁵ | 95% CI above 1 | Met | Yes | 2014Q4 to 2025Q4 | Any eligible target PS/SS report classified as target; reports containing target plus another DMT remain target in this role-misclassification sensitivity |
| PS+SS exposure expansion | Core | Cladribine | Tier A | Active DMTs; PS/SS target definition | 16 | 6359 | 461 | 182582 | 6375 | 183043 | 0.81 (0.50 to 1.33) | 0.406 | 0.406 | 95% CI includes 1 | Met | Yes | 2019Q2 to 2025Q4 | Any eligible target PS/SS report classified as target; reports containing target plus another DMT remain target in this role-misclassification sensitivity |
| Anti-CD20: ocrelizumab omitted | Core | Anti-CD20 core | Tier A | Contemporaneous active DMTs | 17 | 11855 | 253 | 127362 | 11872 | 127615 | 0.49 (0.29 to 0.81) | 0.005 | 0.005 | 95% CI below 1 | Met | Yes | 2017Q2 to 2025Q4 | Leave-one-drug-out; excluded ocrelizumab |
| Anti-CD20: ofatumumab omitted | Core | Anti-CD20 core | Tier A | Contemporaneous active DMTs | 105 | 30817 | 253 | 127362 | 30922 | 127615 | 0.91 (0.69 to 1.18) | 0.466 | 0.466 | 95% CI includes 1 | Met | Yes | 2017Q2 to 2025Q4 | Leave-one-drug-out; excluded ofatumumab |
| S1P: fingolimod omitted | Core | S1P modulators | Tier A | Contemporaneous active DMTs | 7 | 7326 | 367 | 267168 | 7333 | 267535 | 0.51 (0.25 to 1.06) | 0.072 | 0.072 | 95% CI includes 1 | Met | Yes | 2010Q4 to 2025Q4 | Leave-one-drug-out; excluded fingolimod |
| S1P: siponimod omitted | Core | S1P modulators | Tier A | Contemporaneous active DMTs | 146 | 56804 | 367 | 267168 | 56950 | 267535 | 1.90 (1.55 to 2.33) | 4.78 × 10⁻¹⁰ | 4.78 × 10⁻¹⁰ | 95% CI above 1 | Met | Yes | 2010Q4 to 2025Q4 | Leave-one-drug-out; excluded siponimod |
| S1P: ozanimod omitted | Core | S1P modulators | Tier A | Contemporaneous active DMTs | 151 | 56609 | 367 | 267168 | 56760 | 267535 | 1.95 (1.60 to 2.39) | 9.24 × 10⁻¹¹ | 9.24 × 10⁻¹¹ | 95% CI above 1 | Met | Yes | 2010Q4 to 2025Q4 | Leave-one-drug-out; excluded ozanimod |
| S1P: ponesimod omitted | Core | S1P modulators | Tier A | Contemporaneous active DMTs | 152 | 60155 | 367 | 267168 | 60307 | 267535 | 1.78 (1.45 to 2.17) | 1.66 × 10⁻⁸ | 1.66 × 10⁻⁸ | 95% CI above 1 | Met | Yes | 2010Q4 to 2025Q4 | Leave-one-drug-out; excluded ponesimod |
| Anti-CD20 core plus extension | Core | Anti-CD20 core | Tier A | Contemporaneous active DMTs | 132 | 45722 | 253 | 127362 | 45854 | 127615 | 0.84 (0.66 to 1.07) | 0.155 | 0.155 | 95% CI includes 1 | Met | Yes | 2017Q2 to 2025Q4 | Anti-CD20 core plus rituximab/ublituximab extension group |

# Supplementary Table S4. SARS-CoV-2 reporting audit and exclusion sensitivity

Reports containing a COVID-19 or SARS-CoV-2 preferred term were excluded; classic SARS terms without these identifiers were not removed. Estimates are reporting odds ratios, not incidence or mortality risks.

| **Row type** | **DMT mechanism** | **Product** | **Scenario** | **All reports** | **COVID-19/SARS-CoV-2 reports** | **COVID-related Tier A** | **COVID-related Tier A+B** | **Target/reference cases** | **Adjusted aROR (95% CI)** | **BH-adjusted P** | **Interpretation** |
| --- | --- | --- | --- | --- | --- | --- | --- | --- | --- | --- | --- |
| Product-level audit | Alemtuzumab | Alemtuzumab | Audit | 3084 | 16 | 0 | 0 |  |  |  | Reporting-ecology audit |
| Product-level audit | Anti-CD20 core | Ocrelizumab | Audit | 30922 | 7358 | 1 | 6 |  |  |  | Reporting-ecology audit |
| Product-level audit | Anti-CD20 core | Ofatumumab | Audit | 11872 | 520 | 0 | 0 |  |  |  | Reporting-ecology audit |
| Product-level audit | Anti-CD20 extension | Rituximab | Audit | 2366 | 290 | 0 | 1 |  |  |  | Reporting-ecology audit |
| Product-level audit | Anti-CD20 extension | Ublituximab | Audit | 837 | 19 | 0 | 0 |  |  |  | Reporting-ecology audit |
| Product-level audit | Cladribine | Cladribine | Audit | 3077 | 72 | 0 | 0 |  |  |  | Reporting-ecology audit |
| Product-level audit | Interferon-beta/glatiramer acetate | Glatiramer acetate | Audit | 32762 | 127 | 0 | 0 |  |  |  | Reporting-ecology audit |
| Product-level audit | Interferon-beta/glatiramer acetate | Interferon beta | Audit | 112322 | 121 | 0 | 0 |  |  |  | Reporting-ecology audit |
| Product-level audit | Other active/immune DMT | Daclizumab | Audit | 1337 | 0 | 0 | 0 |  |  |  | Reporting-ecology audit |
| Product-level audit | Other active/immune DMT | Dimethyl fumarate | Audit | 67737 | 257 | 0 | 0 |  |  |  | Reporting-ecology audit |
| Product-level audit | Other active/immune DMT | Diroximel fumarate | Audit | 5629 | 166 | 0 | 0 |  |  |  | Reporting-ecology audit |
| Product-level audit | Other active/immune DMT | Mitoxantrone | Audit | 691 | 0 | 0 | 0 |  |  |  | Reporting-ecology audit |
| Product-level audit | Other active/immune DMT | Monomethyl fumarate | Audit | 574 | 6 | 0 | 0 |  |  |  | Reporting-ecology audit |
| Product-level audit | Other active/immune DMT | Teriflunomide | Audit | 26755 | 348 | 0 | 0 |  |  |  | Reporting-ecology audit |
| Product-level audit | Natalizumab | Natalizumab | Audit | 132099 | 450 | 1 | 1 |  |  |  | Reporting-ecology audit |
| Product-level audit | S1P modulators | Fingolimod | Audit | 53117 | 425 | 0 | 1 |  |  |  | Reporting-ecology audit |
| Product-level audit | S1P modulators | Ozanimod | Audit | 3690 | 89 | 0 | 0 |  |  |  | Reporting-ecology audit |
| Product-level audit | S1P modulators | Ponesimod | Audit | 143 | 6 | 0 | 0 |  |  |  | Reporting-ecology audit |
| Product-level audit | S1P modulators | Siponimod | Audit | 3500 | 114 | 0 | 0 |  |  |  | Reporting-ecology audit |
| Adjusted exclusion model | Anti-CD20 core |  | COVID-19/SARS-CoV-2 reports excluded |  |  |  |  | 121/252 | 0.98 (0.77 to 1.26) | 0.884 | Imprecise/null after exclusion |
| Adjusted exclusion model | S1P modulators |  | COVID-19/SARS-CoV-2 reports excluded |  |  |  |  | 152/365 | 1.65 (1.35 to 2.01) | 2.47 × 10⁻⁶ | Positive after exclusion |
| Adjusted exclusion model | Natalizumab |  | COVID-19/SARS-CoV-2 reports excluded |  |  |  |  | 158/381 | 1.27 (1.03 to 1.58) | 0.048 | Positive after exclusion |
| Adjusted exclusion model | Alemtuzumab |  | COVID-19/SARS-CoV-2 reports excluded |  |  |  |  | 35/416 | 3.11 (2.17 to 4.46) | 3.47 × 10⁻⁹ | Positive after exclusion |
| Adjusted exclusion model | Cladribine |  | COVID-19/SARS-CoV-2 reports excluded |  |  |  |  | 5/301 | 0.53 (0.23 to 1.24) | 0.178 | Imprecise/null after exclusion |
| Adjusted exclusion model | Anti-CD20 core |  | Tier A+B; COVID-19/SARS-CoV-2 reports excluded |  |  |  |  | 139/272 | 1.06 (0.84 to 1.34) | 0.620 | Imprecise/null after exclusion |
| Adjusted exclusion model | S1P modulators |  | Tier A+B; COVID-19/SARS-CoV-2 reports excluded |  |  |  |  | 167/404 | 1.67 (1.38 to 2.02) | 3.60 × 10⁻⁷ | Positive after exclusion |
| Adjusted exclusion model | Natalizumab |  | Tier A+B; COVID-19/SARS-CoV-2 reports excluded |  |  |  |  | 170/427 | 1.16 (0.95 to 1.43) | 0.211 | Imprecise/null after exclusion |
| Adjusted exclusion model | Alemtuzumab |  | Tier A+B; COVID-19/SARS-CoV-2 reports excluded |  |  |  |  | 39/457 | 3.21 (2.28 to 4.52) | 1.34 × 10⁻¹⁰ | Positive after exclusion |
| Adjusted exclusion model | Cladribine |  | Tier A+B; COVID-19/SARS-CoV-2 reports excluded |  |  |  |  | 6/332 | 0.58 (0.27 to 1.26) | 0.211 | Imprecise/null after exclusion |

# Supplementary Table S5. S1P product contribution and within-class heterogeneity

Active-comparator models compare each product with contemporaneous non-product MS DMTs; the within-S1P comparison contrasts fingolimod with newer S1P products. The within-S1P comparison is supportive/exploratory because newer products contributed only seven reference Tier A events. PS denotes primary suspect.

| **Row type** | **Product** | **Period** | **Reports** | **Tier A cases** | **Tier A contribution, %** | **Estimate (95% CI)** | **Multiplicity-adjusted P** | **Effect-size statistic** | **Protocol gate** | **Interpretation** |
| --- | --- | --- | --- | --- | --- | --- | --- | --- | --- | --- |
| Product-level active-comparator model | fingolimod | 2010Q4 to 2025Q4 | 53117 | 145 | 95.4 | 2.15 (1.75 to 2.64) | 1.40 × 10⁻¹² |  | Met | Signal driver |
| Product-level active-comparator model | siponimod | 2019Q2 to 2025Q4 | 3500 | 6 | 3.9 | 0.83 (0.38 to 1.82) | 0.777 |  | Met | Imprecise/information limited |
| Product-level active-comparator model | ozanimod | 2020Q3 to 2025Q4 | 3690 | 1 | 0.7 | 0.22 (0.05 to 1.10) | 0.129 |  | Met | Imprecise/information limited |
| Product-level active-comparator model | ponesimod | 2021Q2 to 2025Q4 | 143 | 0 | 0 | 1.49 (0.09 to 23.55) | 0.777 |  | Met | Imprecise/information limited |
| Four-product common-period heterogeneity | All four S1P products | 2021Q2 to 2025Q4 | 12413 | 75 |  |  | 1.00 × 10⁻⁵ | Cramer's V=0.063 | Met | Statistically heterogeneous; small effect size |
| Within-S1P supportive comparison | fingolimod vs newer S1P products | 2019Q2 to 2025Q4 | 25131 | 96 |  | 10.59 (5.11 to 25.27) | 1.16 × 10⁻⁹ |  | Not met | Exploratory: only seven newer-product reference events |

# Supplementary Table S6. Full pathogen-composition models and global-model diagnostics

For one-versus-rest rows, analytic N and pathogen-class counts refer to the pooled target-plus-reference comparison set. Seven mixed-pathogen reports were pooled with other/unspecified in the global model; cladribine was excluded from the omnibus model because Tier A+B n=6. Cramér’s V is the complementary unadjusted effect-size summary. Sparse protozoan/parasite cells were not modeled.

| **Row type** | **DMT mechanism** | **Pathogen category** | **Pooled target-plus-reference analytic reports, n** | **Pooled pathogen-class reports, n** | **Estimate/test statistic** | **df** | **Adjusted P** | **Effect size** | **Numerical diagnostic** | **Interpretation** |
| --- | --- | --- | --- | --- | --- | --- | --- | --- | --- | --- |
| One-vs-rest composition model | Anti-CD20 core | Virus | 414 | 178 | 4.24 (2.56 to 7.02) |  | 7.86 × 10⁻⁸ |  | Binary one-versus-rest model | Composition model |
| One-vs-rest composition model | Anti-CD20 core | Fungus | 414 | 97 | 0.010 (0.003 to 0.041) |  | 5.34 × 10⁻¹⁰ |  | Binary one-versus-rest model | Composition model |
| One-vs-rest composition model | Anti-CD20 core | Bacterium | 414 | 68 | 1.55 (0.83 to 2.90) |  | 0.231 |  | Binary one-versus-rest model | Composition model |
| One-vs-rest composition model | Anti-CD20 core | Protozoan/parasite | 414 | 2 | NE |  | NE |  | Below binary downgrade threshold | Below class-size gate |
| One-vs-rest composition model | S1P modulators | Virus | 573 | 259 | 0.22 (0.14 to 0.35) |  | 5.60 × 10⁻¹⁰ |  | Binary one-versus-rest model | Composition model |
| One-vs-rest composition model | S1P modulators | Fungus | 573 | 114 | 29.09 (15.77 to 53.64) |  | 5.52 × 10⁻²⁶ |  | Binary one-versus-rest model | Composition model |
| One-vs-rest composition model | S1P modulators | Bacterium | 573 | 102 | 0.25 (0.13 to 0.49) |  | 1.43 × 10⁻⁴ |  | Binary one-versus-rest model | Composition model |
| One-vs-rest composition model | S1P modulators | Protozoan/parasite | 573 | 3 | NE |  | NE |  | Below binary downgrade threshold | Below class-size gate |
| One-vs-rest composition model | Natalizumab | Virus | 605 | 281 | 2.03 (1.26 to 3.26) |  | 0.006 |  | Binary one-versus-rest model | Composition model |
| One-vs-rest composition model | Natalizumab | Fungus | 605 | 114 | 0.21 (0.09 to 0.48) |  | 6.23 × 10⁻⁴ |  | Binary one-versus-rest model | Composition model |
| One-vs-rest composition model | Natalizumab | Bacterium | 605 | 105 | 1.01 (0.57 to 1.81) |  | 0.980 |  | Binary one-versus-rest model | Composition model |
| One-vs-rest composition model | Natalizumab | Protozoan/parasite | 605 | 5 | NE |  | NE |  | Below binary downgrade threshold | Below class-size gate |
| One-vs-rest composition model | Alemtuzumab | Virus | 504 | 213 | 0.26 (0.11 to 0.64) |  | 0.006 |  | Binary one-versus-rest model | Composition model |
| One-vs-rest composition model | Alemtuzumab | Fungus | 504 | 111 | 0.29 (0.08 to 1.11) |  | 0.105 |  | Binary one-versus-rest model | Composition model |
| One-vs-rest composition model | Alemtuzumab | Bacterium | 504 | 96 | 4.99 (2.27 to 11.01) |  | 1.66 × 10⁻⁴ |  | Binary one-versus-rest model | Composition model |
| One-vs-rest composition model | Alemtuzumab | Protozoan/parasite | 504 | 2 | NE |  | NE |  | Below binary downgrade threshold | Below class-size gate |
| One-vs-rest composition model | Cladribine | Virus | 284 | 135 | 1.02 (0.18 to 5.71) |  | 0.980 |  | Binary one-versus-rest model | Composition model |
| One-vs-rest composition model | Cladribine | Fungus | 284 | 71 | 0.21 (0.01 to 5.49) |  | 0.439 |  | Binary one-versus-rest model | Composition model |
| One-vs-rest composition model | Cladribine | Bacterium | 284 | 35 | 2.11 (0.27 to 16.54) |  | 0.551 |  | Binary one-versus-rest model | Composition model |
| One-vs-rest composition model | Cladribine | Protozoan/parasite | 284 | 0 | NE |  | NE |  | Below binary downgrade threshold | Below class-size gate |
| Global multinomial heterogeneity | Anti-CD20; S1P; natalizumab; alemtuzumab | Virus;fungus;bacterium;other mixed unspecified | 523 |  | LRT=210.59 | 9 | 9.99 × 10⁻⁴ | Cramer's V=0.415 | Hessian condition=2714; 1000/1000 bootstrap replicates usable | Cladribine excluded from omnibus because Tier A+B n=6; Mixed pathogen reports (n=7) pooled with other/unspecified; age and MS-subtype sparse levels consolidated before modeling |

# Supplementary Table S7. Four-cell data and adjusted same-pathogen CNS case-case models

H1=anti-CD20–enterovirus; H2=S1P–Cryptococcus; H3=natalizumab–HSV/VZV; H4=alemtuzumab–Listeria. Only H3 met the adjusted-model reference-event gate. MH-ROR denotes the calendar-year Mantel–Haenszel reporting odds ratio; NE denotes not estimable.

| **Hypothesis** | **DMT mechanism** | **Pathogen** | **Target CNS cases (a)** | **Target non-CNS cases (b)** | **Reference CNS cases (c)** | **Reference non-CNS cases (d)** | **Calendar-year MH-ROR (95% CI)** | **MH Holm P** | **Adjusted Firth aROR (95% CI)** | **Adjusted Holm P** | **Adjusted-model gate** | **Gate reason** | **Converged** |
| --- | --- | --- | --- | --- | --- | --- | --- | --- | --- | --- | --- | --- | --- |
| H1 | Anti-CD20 core | Enterovirus | 14 | 19 | 1 | 5 | 1.48 (0.16 to 13.93) | 1.000 | Not modeled | NE | Not met | Total events: 15; reference events: 1 | No/not fitted |
| H2 | S1P modulators | Cryptococcus | 91 | 80 | 8 | 4 | 0.89 (0.25 to 3.18) | 1.000 | Not modeled | NE | Not met | Total events: 99; reference events: 8 | No/not fitted |
| H3 | Natalizumab | HSV/VZV | 62 | 1601 | 78 | 3616 | 2.45 (1.59 to 3.77) | 0.003 | 2.80 (1.80 to 4.38) | 5.81 × 10⁻⁶ | Met | Total events: 140; reference events: 78 | Yes |
| H4 | Alemtuzumab | Listeria | 23 | 30 | 6 | 7 | 0.82 (0.21 to 3.24) | 1.000 | Not modeled | NE | Not met | Total events: 29; reference events: 6 | No/not fitted |

# Supplementary Table S8. Positive controls and alternative estimands/comparators

The estimator-specific P column identifies the estimator linked to each displayed P value. MH-ROR denotes the calendar-year Mantel–Haenszel reporting odds ratio; aROR denotes the covariate-adjusted Jeffreys-prior Firth reporting odds ratio. The interferon-beta/glatiramer-acetate comparator is restricted to that mechanism group; the all-other-mapped-MS-DMT comparator includes all mapped non-target DMT reports in the same launch-defined window. Comparator analyses are secondary and do not replace the locked primary contrast.

| **Row type** | **Analysis** | **DMT mechanism** | **Target/reference cases** | **Crude ROR (95% CI)** | **MH-ROR (95% CI)** | **Adjusted aROR (95% CI)** | **Estimator-specific P** | **Status** | **Note** |
| --- | --- | --- | --- | --- | --- | --- | --- | --- | --- |
| Positive control | Natalizumab–PML | Natalizumab | 1475/355 | 6.73 (5.99 to 7.56) | 15.48 (13.60 to 17.63) |  | Crude P=1.04 × 10⁻³⁰⁰ | Signal threshold met | Internal validity control |
| Positive control | S1P–herpes infection | S1P modulators | 990/4046 | 1.08 (1.01 to 1.16) | 1.05 (0.98 to 1.13) |  | Crude P=0.024 | Signal threshold not met | Internal validity control |
| Alternative estimand/comparator | Infection-report denominator | Anti-CD20 core | 122/253 |  |  | 0.26 (0.20 to 0.34) | Adjusted P=2.68 × 10⁻²³ | 95% CI below 1 | Tier A CNS infection versus other non-PML infection-related reports |
| Alternative estimand/comparator | Infection-report denominator | S1P modulators | 152/367 |  |  | 2.52 (2.05 to 3.10) | Adjusted P=2.26 × 10⁻¹⁸ | 95% CI above 1 | Tier A CNS infection versus other non-PML infection-related reports |
| Alternative estimand/comparator | Infection-report denominator | Natalizumab | 159/382 |  |  | 1.58 (1.24 to 2.01) | Adjusted P=1.89 × 10⁻⁴ | 95% CI above 1 | Tier A CNS infection versus other non-PML infection-related reports |
| Alternative estimand/comparator | Infection-report denominator | Alemtuzumab | 35/418 |  |  | 1.46 (1.00 to 2.14) | Adjusted P=0.048 | 95% CI above 1 | Tier A CNS infection versus other non-PML infection-related reports |
| Alternative estimand/comparator | Infection-report denominator | Cladribine | 5/303 |  |  | 0.79 (0.34 to 1.87) | Adjusted P=0.596 | 95% CI includes 1 | Tier A CNS infection versus other non-PML infection-related reports |
| Alternative estimand/comparator | Interferon-beta/glatiramer acetate comparator | Anti-CD20 core | 122/19 |  |  | 6.25 (3.59 to 10.87) | Adjusted P=8.88 × 10⁻¹¹ | 95% CI above 1 | Low-immunosuppression comparator |
| Alternative estimand/comparator | Interferon-beta/glatiramer acetate comparator | S1P modulators | 152/66 |  |  | 3.94 (2.88 to 5.39) | Adjusted P=8.66 × 10⁻¹⁸ | 95% CI above 1 | Low-immunosuppression comparator |
| Alternative estimand/comparator | Interferon-beta/glatiramer acetate comparator | Natalizumab | 159/105 |  |  | 2.39 (1.84 to 3.09) | Adjusted P=4.89 × 10⁻¹¹ | 95% CI above 1 | Low-immunosuppression comparator |
| Alternative estimand/comparator | Interferon-beta/glatiramer acetate comparator | Alemtuzumab | 35/35 |  |  | 11.52 (6.96 to 19.08) | Adjusted P=2.10 × 10⁻²¹ | 95% CI above 1 | Low-immunosuppression comparator |
| Alternative estimand/comparator | Interferon-beta/glatiramer acetate comparator | Cladribine | 5/12 |  |  | NE | Adjusted P=NE | Not estimable | Low-immunosuppression comparator |
| Alternative estimand/comparator | All other MS-DMT comparator | Anti-CD20 core | 122/282 |  |  | 1.00 (0.78 to 1.29) | Adjusted P=0.994 | 95% CI includes 1 | All other mapped MS-DMT comparator |
| Alternative estimand/comparator | All other MS-DMT comparator | S1P modulators | 152/444 |  |  | 2.13 (1.75 to 2.58) | Adjusted P=2.00 × 10⁻¹⁴ | 95% CI above 1 | All other mapped MS-DMT comparator |
| Alternative estimand/comparator | All other MS-DMT comparator | Natalizumab | 159/498 |  |  | 1.50 (1.23 to 1.82) | Adjusted P=4.17 × 10⁻⁵ | 95% CI above 1 | All other mapped MS-DMT comparator |
| Alternative estimand/comparator | All other MS-DMT comparator | Alemtuzumab | 35/464 |  |  | 3.96 (2.77 to 5.66) | Adjusted P=4.17 × 10⁻¹⁴ | 95% CI above 1 | All other mapped MS-DMT comparator |
| Alternative estimand/comparator | All other MS-DMT comparator | Cladribine | 5/324 |  |  | 0.66 (0.28 to 1.52) | Adjusted P=0.327 | 95% CI includes 1 | All other mapped MS-DMT comparator |
| Alternative estimand/comparator | PS+SS exposure expansion | Anti-CD20 core | 177/398 |  |  | 0.77 (0.62 to 0.95) | Adjusted P=0.015 | 95% CI below 1 | Any eligible target PS/SS report classified as target; reports containing target plus another DMT remain target in this role-misclassification sensitivity |
| Alternative estimand/comparator | PS+SS exposure expansion | S1P modulators | 199/571 |  |  | 1.65 (1.39 to 1.96) | Adjusted P=9.78 × 10⁻⁹ | 95% CI above 1 | Any eligible target PS/SS report classified as target; reports containing target plus another DMT remain target in this role-misclassification sensitivity |
| Alternative estimand/comparator | PS+SS exposure expansion | Natalizumab | 242/553 |  |  | 1.62 (1.36 to 1.93) | Adjusted P=4.83 × 10⁻⁸ | 95% CI above 1 | Any eligible target PS/SS report classified as target; reports containing target plus another DMT remain target in this role-misclassification sensitivity |
| Alternative estimand/comparator | PS+SS exposure expansion | Alemtuzumab | 49/635 |  |  | 1.92 (1.42 to 2.60) | Adjusted P=2.22 × 10⁻⁵ | 95% CI above 1 | Any eligible target PS/SS report classified as target; reports containing target plus another DMT remain target in this role-misclassification sensitivity |
| Alternative estimand/comparator | PS+SS exposure expansion | Cladribine | 16/461 |  |  | 0.81 (0.50 to 1.33) | Adjusted P=0.406 | 95% CI includes 1 | Any eligible target PS/SS report classified as target; reports containing target plus another DMT remain target in this role-misclassification sensitivity |

# Supplementary Table S9. Serious outcomes, time-to-onset summaries, and availability diagnostics

Serious-outcome and death values are descriptive report-level flags among Tier A+B infection reports only and are not comparable with the all-report proportions in Table 1. DE=death; LT=life-threatening; HO=hospitalization; DS=disability; CA=congenital anomaly; RI=required intervention; OT=other serious outcome. TTO uses complete, logically valid dates only and is descriptive; no censoring, incidence, or survival-risk interpretation is made.

| **Row type** | **DMT/product/group** | **Metric** | **N** | **Events** | **Value/estimate** | **Additional result** | **Interpretation** |
| --- | --- | --- | --- | --- | --- | --- | --- |
| Serious outcome description | Interferon-beta/glatiramer acetate / Interferon beta | Any reported serious outcome | 124 | 124 | 100.0% (97.1 to 100.0) |  | Descriptive proportion among reports |
| Serious outcome description | Interferon-beta/glatiramer acetate / Glatiramer acetate | Any reported serious outcome | 16 | 16 | 100.0% (79.4 to 100.0) |  | Descriptive proportion among reports |
| Serious outcome description | Natalizumab / Natalizumab | Any reported serious outcome | 171 | 159 | 93.0% (88.1 to 96.3) |  | Descriptive proportion among reports |
| Serious outcome description | S1P modulators / Fingolimod | Any reported serious outcome | 161 | 160 | 99.4% (96.6 to 100.0) |  | Descriptive proportion among reports |
| Serious outcome description | Other active/immune DMT / Dimethyl fumarate | Any reported serious outcome | 50 | 49 | 98.0% (89.4 to 99.9) |  | Descriptive proportion among reports |
| Serious outcome description | Other active/immune DMT / Teriflunomide | Any reported serious outcome | 17 | 17 | 100.0% (80.5 to 100.0) |  | Descriptive proportion among reports |
| Serious outcome description | Alemtuzumab / Alemtuzumab | Any reported serious outcome | 39 | 39 | 100.0% (91.0 to 100.0) |  | Descriptive proportion among reports |
| Serious outcome description | Anti-CD20 extension / Rituximab | Any reported serious outcome | 12 | 12 | 100.0% (73.5 to 100.0) |  | Descriptive proportion among reports |
| Serious outcome description | Anti-CD20 core / Ocrelizumab | Any reported serious outcome | 123 | 120 | 97.6% (93.0 to 99.5) |  | Descriptive proportion among reports |
| Serious outcome description | Other active/immune DMT / Daclizumab | Any reported serious outcome | 3 | 3 | 100.0% (29.2 to 100.0) |  | Descriptive proportion among reports |
| Serious outcome description | S1P modulators / Siponimod | Any reported serious outcome | 6 | 6 | 100.0% (54.1 to 100.0) |  | Descriptive proportion among reports |
| Serious outcome description | Cladribine / Cladribine | Any reported serious outcome | 6 | 6 | 100.0% (54.1 to 100.0) |  | Descriptive proportion among reports |
| Serious outcome description | Other active/immune DMT / Diroximel fumarate | Any reported serious outcome | 5 | 4 | 80.0% (28.4 to 99.5) |  | Descriptive proportion among reports |
| Serious outcome description | S1P modulators / Ozanimod | Any reported serious outcome | 1 | 1 | 100.0% (2.5 to 100.0) |  | Descriptive proportion among reports |
| Serious outcome description | Anti-CD20 core / Ofatumumab | Any reported serious outcome | 22 | 22 | 100.0% (84.6 to 100.0) |  | Descriptive proportion among reports |
| Serious outcome description | Other active/immune DMT / Monomethyl fumarate | Any reported serious outcome | 1 | 1 | 100.0% (2.5 to 100.0) |  | Descriptive proportion among reports |
| Serious outcome description | Interferon-beta/glatiramer acetate / Interferon beta | Reported death | 124 | 13 | 10.5% (5.7 to 17.3) |  | Descriptive proportion among reports |
| Serious outcome description | Interferon-beta/glatiramer acetate / Glatiramer acetate | Reported death | 16 | 1 | 6.2% (0.2 to 30.2) |  | Descriptive proportion among reports |
| Serious outcome description | Natalizumab / Natalizumab | Reported death | 171 | 10 | 5.8% (2.8 to 10.5) |  | Descriptive proportion among reports |
| Serious outcome description | S1P modulators / Fingolimod | Reported death | 161 | 20 | 12.4% (7.8 to 18.5) |  | Descriptive proportion among reports |
| Serious outcome description | Other active/immune DMT / Dimethyl fumarate | Reported death | 50 | 3 | 6.0% (1.3 to 16.5) |  | Descriptive proportion among reports |
| Serious outcome description | Other active/immune DMT / Teriflunomide | Reported death | 17 | 2 | 11.8% (1.5 to 36.4) |  | Descriptive proportion among reports |
| Serious outcome description | Alemtuzumab / Alemtuzumab | Reported death | 39 | 5 | 12.8% (4.3 to 27.4) |  | Descriptive proportion among reports |
| Serious outcome description | Anti-CD20 extension / Rituximab | Reported death | 12 | 2 | 16.7% (2.1 to 48.4) |  | Descriptive proportion among reports |
| Serious outcome description | Anti-CD20 core / Ocrelizumab | Reported death | 123 | 9 | 7.3% (3.4 to 13.4) |  | Descriptive proportion among reports |
| Serious outcome description | Other active/immune DMT / Daclizumab | Reported death | 3 | 0 | 0.0% (0.0 to 70.8) |  | Descriptive proportion among reports |
| Serious outcome description | S1P modulators / Siponimod | Reported death | 6 | 0 | 0.0% (0.0 to 45.9) |  | Descriptive proportion among reports |
| Serious outcome description | Cladribine / Cladribine | Reported death | 6 | 0 | 0.0% (0.0 to 45.9) |  | Descriptive proportion among reports |
| Serious outcome description | Other active/immune DMT / Diroximel fumarate | Reported death | 5 | 0 | 0.0% (0.0 to 52.2) |  | Descriptive proportion among reports |
| Serious outcome description | S1P modulators / Ozanimod | Reported death | 1 | 0 | 0.0% (0.0 to 97.5) |  | Descriptive proportion among reports |
| Serious outcome description | Anti-CD20 core / Ofatumumab | Reported death | 22 | 1 | 4.5% (0.1 to 22.8) |  | Descriptive proportion among reports |
| Serious outcome description | Other active/immune DMT / Monomethyl fumarate | Reported death | 1 | 0 | 0.0% (0.0 to 97.5) |  | Descriptive proportion among reports |
| Serious outcome description | Interferon-beta/glatiramer acetate / Interferon beta | Reported life-threatening event | 124 | 13 | 10.5% (5.7 to 17.3) |  | Descriptive proportion among reports |
| Serious outcome description | Interferon-beta/glatiramer acetate / Glatiramer acetate | Reported life-threatening event | 16 | 0 | 0.0% (0.0 to 20.6) |  | Descriptive proportion among reports |
| Serious outcome description | Natalizumab / Natalizumab | Reported life-threatening event | 171 | 10 | 5.8% (2.8 to 10.5) |  | Descriptive proportion among reports |
| Serious outcome description | S1P modulators / Fingolimod | Reported life-threatening event | 161 | 29 | 18.0% (12.4 to 24.8) |  | Descriptive proportion among reports |
| Serious outcome description | Other active/immune DMT / Dimethyl fumarate | Reported life-threatening event | 50 | 2 | 4.0% (0.5 to 13.7) |  | Descriptive proportion among reports |
| Serious outcome description | Other active/immune DMT / Teriflunomide | Reported life-threatening event | 17 | 1 | 5.9% (0.1 to 28.7) |  | Descriptive proportion among reports |
| Serious outcome description | Alemtuzumab / Alemtuzumab | Reported life-threatening event | 39 | 5 | 12.8% (4.3 to 27.4) |  | Descriptive proportion among reports |
| Serious outcome description | Anti-CD20 extension / Rituximab | Reported life-threatening event | 12 | 0 | 0.0% (0.0 to 26.5) |  | Descriptive proportion among reports |
| Serious outcome description | Anti-CD20 core / Ocrelizumab | Reported life-threatening event | 123 | 10 | 8.1% (4.0 to 14.4) |  | Descriptive proportion among reports |
| Serious outcome description | Other active/immune DMT / Daclizumab | Reported life-threatening event | 3 | 1 | 33.3% (0.8 to 90.6) |  | Descriptive proportion among reports |
| Serious outcome description | S1P modulators / Siponimod | Reported life-threatening event | 6 | 1 | 16.7% (0.4 to 64.1) |  | Descriptive proportion among reports |
| Serious outcome description | Cladribine / Cladribine | Reported life-threatening event | 6 | 0 | 0.0% (0.0 to 45.9) |  | Descriptive proportion among reports |
| Serious outcome description | Other active/immune DMT / Diroximel fumarate | Reported life-threatening event | 5 | 2 | 40.0% (5.3 to 85.3) |  | Descriptive proportion among reports |
| Serious outcome description | S1P modulators / Ozanimod | Reported life-threatening event | 1 | 0 | 0.0% (0.0 to 97.5) |  | Descriptive proportion among reports |
| Serious outcome description | Anti-CD20 core / Ofatumumab | Reported life-threatening event | 22 | 5 | 22.7% (7.8 to 45.4) |  | Descriptive proportion among reports |
| Serious outcome description | Other active/immune DMT / Monomethyl fumarate | Reported life-threatening event | 1 | 0 | 0.0% (0.0 to 97.5) |  | Descriptive proportion among reports |
| Serious outcome description | Interferon-beta/glatiramer acetate / Interferon beta | Reported hospitalization | 124 | 105 | 84.7% (77.1 to 90.5) |  | Descriptive proportion among reports |
| Serious outcome description | Interferon-beta/glatiramer acetate / Glatiramer acetate | Reported hospitalization | 16 | 11 | 68.8% (41.3 to 89.0) |  | Descriptive proportion among reports |
| Serious outcome description | Natalizumab / Natalizumab | Reported hospitalization | 171 | 129 | 75.4% (68.3 to 81.7) |  | Descriptive proportion among reports |
| Serious outcome description | S1P modulators / Fingolimod | Reported hospitalization | 161 | 124 | 77.0% (69.7 to 83.3) |  | Descriptive proportion among reports |
| Serious outcome description | Other active/immune DMT / Dimethyl fumarate | Reported hospitalization | 50 | 31 | 62.0% (47.2 to 75.3) |  | Descriptive proportion among reports |
| Serious outcome description | Other active/immune DMT / Teriflunomide | Reported hospitalization | 17 | 6 | 35.3% (14.2 to 61.7) |  | Descriptive proportion among reports |
| Serious outcome description | Alemtuzumab / Alemtuzumab | Reported hospitalization | 39 | 31 | 79.5% (63.5 to 90.7) |  | Descriptive proportion among reports |
| Serious outcome description | Anti-CD20 extension / Rituximab | Reported hospitalization | 12 | 4 | 33.3% (9.9 to 65.1) |  | Descriptive proportion among reports |
| Serious outcome description | Anti-CD20 core / Ocrelizumab | Reported hospitalization | 123 | 71 | 57.7% (48.5 to 66.6) |  | Descriptive proportion among reports |
| Serious outcome description | Other active/immune DMT / Daclizumab | Reported hospitalization | 3 | 3 | 100.0% (29.2 to 100.0) |  | Descriptive proportion among reports |
| Serious outcome description | S1P modulators / Siponimod | Reported hospitalization | 6 | 4 | 66.7% (22.3 to 95.7) |  | Descriptive proportion among reports |
| Serious outcome description | Cladribine / Cladribine | Reported hospitalization | 6 | 5 | 83.3% (35.9 to 99.6) |  | Descriptive proportion among reports |
| Serious outcome description | Other active/immune DMT / Diroximel fumarate | Reported hospitalization | 5 | 0 | 0.0% (0.0 to 52.2) |  | Descriptive proportion among reports |
| Serious outcome description | S1P modulators / Ozanimod | Reported hospitalization | 1 | 1 | 100.0% (2.5 to 100.0) |  | Descriptive proportion among reports |
| Serious outcome description | Anti-CD20 core / Ofatumumab | Reported hospitalization | 22 | 18 | 81.8% (59.7 to 94.8) |  | Descriptive proportion among reports |
| Serious outcome description | Other active/immune DMT / Monomethyl fumarate | Reported hospitalization | 1 | 0 | 0.0% (0.0 to 97.5) |  | Descriptive proportion among reports |
| Serious outcome description | Interferon-beta/glatiramer acetate / Interferon beta | Reported disability | 124 | 4 | 3.2% (0.9 to 8.1) |  | Descriptive proportion among reports |
| Serious outcome description | Interferon-beta/glatiramer acetate / Glatiramer acetate | Reported disability | 16 | 1 | 6.2% (0.2 to 30.2) |  | Descriptive proportion among reports |
| Serious outcome description | Natalizumab / Natalizumab | Reported disability | 171 | 4 | 2.3% (0.6 to 5.9) |  | Descriptive proportion among reports |
| Serious outcome description | S1P modulators / Fingolimod | Reported disability | 161 | 12 | 7.5% (3.9 to 12.7) |  | Descriptive proportion among reports |
| Serious outcome description | Other active/immune DMT / Dimethyl fumarate | Reported disability | 50 | 1 | 2.0% (0.1 to 10.6) |  | Descriptive proportion among reports |
| Serious outcome description | Other active/immune DMT / Teriflunomide | Reported disability | 17 | 1 | 5.9% (0.1 to 28.7) |  | Descriptive proportion among reports |
| Serious outcome description | Alemtuzumab / Alemtuzumab | Reported disability | 39 | 3 | 7.7% (1.6 to 20.9) |  | Descriptive proportion among reports |
| Serious outcome description | Anti-CD20 extension / Rituximab | Reported disability | 12 | 0 | 0.0% (0.0 to 26.5) |  | Descriptive proportion among reports |
| Serious outcome description | Anti-CD20 core / Ocrelizumab | Reported disability | 123 | 5 | 4.1% (1.3 to 9.2) |  | Descriptive proportion among reports |
| Serious outcome description | Other active/immune DMT / Daclizumab | Reported disability | 3 | 0 | 0.0% (0.0 to 70.8) |  | Descriptive proportion among reports |
| Serious outcome description | S1P modulators / Siponimod | Reported disability | 6 | 1 | 16.7% (0.4 to 64.1) |  | Descriptive proportion among reports |
| Serious outcome description | Cladribine / Cladribine | Reported disability | 6 | 0 | 0.0% (0.0 to 45.9) |  | Descriptive proportion among reports |
| Serious outcome description | Other active/immune DMT / Diroximel fumarate | Reported disability | 5 | 0 | 0.0% (0.0 to 52.2) |  | Descriptive proportion among reports |
| Serious outcome description | S1P modulators / Ozanimod | Reported disability | 1 | 0 | 0.0% (0.0 to 97.5) |  | Descriptive proportion among reports |
| Serious outcome description | Anti-CD20 core / Ofatumumab | Reported disability | 22 | 4 | 18.2% (5.2 to 40.3) |  | Descriptive proportion among reports |
| Serious outcome description | Other active/immune DMT / Monomethyl fumarate | Reported disability | 1 | 0 | 0.0% (0.0 to 97.5) |  | Descriptive proportion among reports |
| Serious outcome description | Interferon-beta/glatiramer acetate / Interferon beta | Other reported serious outcome | 124 | 43 | 34.7% (26.4 to 43.7) |  | Descriptive proportion among reports |
| Serious outcome description | Interferon-beta/glatiramer acetate / Glatiramer acetate | Other reported serious outcome | 16 | 12 | 75.0% (47.6 to 92.7) |  | Descriptive proportion among reports |
| Serious outcome description | Natalizumab / Natalizumab | Other reported serious outcome | 171 | 61 | 35.7% (28.5 to 43.3) |  | Descriptive proportion among reports |
| Serious outcome description | S1P modulators / Fingolimod | Other reported serious outcome | 161 | 103 | 64.0% (56.0 to 71.4) |  | Descriptive proportion among reports |
| Serious outcome description | Other active/immune DMT / Dimethyl fumarate | Other reported serious outcome | 50 | 23 | 46.0% (31.8 to 60.7) |  | Descriptive proportion among reports |
| Serious outcome description | Other active/immune DMT / Teriflunomide | Other reported serious outcome | 17 | 14 | 82.4% (56.6 to 96.2) |  | Descriptive proportion among reports |
| Serious outcome description | Alemtuzumab / Alemtuzumab | Other reported serious outcome | 39 | 22 | 56.4% (39.6 to 72.2) |  | Descriptive proportion among reports |
| Serious outcome description | Anti-CD20 extension / Rituximab | Other reported serious outcome | 12 | 7 | 58.3% (27.7 to 84.8) |  | Descriptive proportion among reports |
| Serious outcome description | Anti-CD20 core / Ocrelizumab | Other reported serious outcome | 123 | 60 | 48.8% (39.7 to 58.0) |  | Descriptive proportion among reports |
| Serious outcome description | Other active/immune DMT / Daclizumab | Other reported serious outcome | 3 | 1 | 33.3% (0.8 to 90.6) |  | Descriptive proportion among reports |
| Serious outcome description | S1P modulators / Siponimod | Other reported serious outcome | 6 | 5 | 83.3% (35.9 to 99.6) |  | Descriptive proportion among reports |
| Serious outcome description | Cladribine / Cladribine | Other reported serious outcome | 6 | 1 | 16.7% (0.4 to 64.1) |  | Descriptive proportion among reports |
| Serious outcome description | Other active/immune DMT / Diroximel fumarate | Other reported serious outcome | 5 | 3 | 60.0% (14.7 to 94.7) |  | Descriptive proportion among reports |
| Serious outcome description | S1P modulators / Ozanimod | Other reported serious outcome | 1 | 0 | 0.0% (0.0 to 97.5) |  | Descriptive proportion among reports |
| Serious outcome description | Anti-CD20 core / Ofatumumab | Other reported serious outcome | 22 | 9 | 40.9% (20.7 to 63.6) |  | Descriptive proportion among reports |
| Serious outcome description | Other active/immune DMT / Monomethyl fumarate | Other reported serious outcome | 1 | 1 | 100.0% (2.5 to 100.0) |  | Descriptive proportion among reports |
| TTO summary | Interferon-beta/glatiramer acetate / Glatiramer acetate | Time to onset | 2 |  | Median 728 days (IQR 546 to 910) | Bootstrap median 95% CI NA to NA days | Complete, logically valid dates only |
| TTO summary | Interferon-beta/glatiramer acetate / Interferon beta | Time to onset | 65 |  | Median 317 days (IQR 104 to 1188) | Bootstrap median 95% CI 206 to 836 days | Complete, logically valid dates only |
| TTO summary | Natalizumab / Natalizumab | Time to onset | 34 |  | Median 373 days (IQR 147 to 793) | Bootstrap median 95% CI 188 to 552 days | Complete, logically valid dates only |
| TTO summary | S1P modulators / Fingolimod | Time to onset | 2 |  | Median 254 days (IQR 218 to 290) | Bootstrap median 95% CI NA to NA days | Complete, logically valid dates only |
| TTO availability bias | TTO available | Availability-group profile | 103 |  | Age known 98.1%; female 76.7%; HCP 42.7% | Serious 97.1%; death 7.8% | Demonstrates selection in the analyzable TTO subset |
| TTO availability bias | TTO unavailable | Availability-group profile | 654 |  | Age known 73.9%; female 64.5%; HCP 63.6% | Serious 97.7%; death 8.9% | Demonstrates selection in the analyzable TTO subset |
| Exploratory Weibull diagnostic | Interferon-beta/glatiramer acetate | Weibull fit | 66 |  | Shape 0.833; scale 641.1 | Convergence: converged; uncensored intercept-only Weibull MLE; no hazard interpretation | Exploratory only; no survival-risk interpretation |
| Exploratory Weibull diagnostic | Natalizumab | Weibull fit | 34 |  | Shape 1.218; scale 494.2 | Convergence: converged; uncensored intercept-only Weibull MLE; no hazard interpretation | Exploratory only; no survival-risk interpretation |
| Exploratory Weibull diagnostic | S1P modulators | Weibull fit | 2 |  | Not modeled | Convergence: model not fitted (<30 events) | Exploratory only; no survival-risk interpretation |

## Supplementary Table S10. Full screening dictionary audit and endpoint roles

| Dictionary component | PTs | Role | Analysis use |
| --- | --- | --- | --- |
| A1 | 85 | Direct, pathogen-specific CNS infection terms | Tier A |
| A2 | 27 | Explicitly infectious CNS syndrome terms | Tier A |
| Tier C | 10 | Ambiguous CNS syndrome terms | Exploratory only |
| Non-infectious exclusion | 34 | Drug-induced, immune-mediated, aseptic, or other non-infectious mimics | Excluded |
| PML/JCV exclusion | 7 | Progressive multifocal leukoencephalopathy or JC-virus terms | Excluded from target; audit/control |
| Additional pathogen-evidence terms | 1464 | Extracranial or non-CNS pathogen-coded terms | Tier B/context/E2 |
| Immune/neurovascular proxy terms | 21 | Context terms not used as infection evidence | Context only |
| Total dictionary | 1648 | All MedDRA v29 preferred terms in the locked codebook | Complete dictionary |
| Broad infection flag union | 1580 | Pathogen-evidence terms plus A1/A2 before PML/JCV and non-infectious endpoint exclusions; 1,576 after removal of four pathogen-coded PML/JCV PTs | Sensitivity/context only |

The complete 1,648-term dictionary is provided as Supplementary Data S1A (Excel workbook). Supplementary Table S1 lists the 163 terms that directly determine CNS tier or explicit exclusion. The broad infection flag is a dictionary-based sensitivity construct and is not interpreted as an exhaustive ascertainment of all infections.

# Supplementary Figure S1. Adjusted sensitivity and leave-one-drug-out matrix

(A) Adjusted aRORs across prespecified endpoint, denominator, reporter, temporal, carryover, restriction, and exposure-definition analyses. (B) Product-decomposition analyses omitting individual anti-CD20 or S1P products, together with the anti-CD20 core-plus-extension definition. All analyses are shown regardless of direction or statistical significance.

# Supplementary Figure S2. Bias-directed sensitivity analyses show comparator-dependent magnitude

Adjusted estimates under the locked active-comparator specification, SARS-CoV-2-report exclusion, expanded PS/SS exposure definition, all-other-MS-DMT comparator, and interferon-beta/glatiramer-acetate comparator. Comparator changes probe confounding and reporting structure; they are not alternative primary analyses.

# Supplementary Figure S3. Internal validity checks and cross-scenario stability

(A) Natalizumab–PML and S1P–herpes-infection positive-control analyses. (B) Minimum, maximum, median, and locked-primary point estimates across core adjusted sensitivity scenarios. Ranges summarize point estimates rather than confidence intervals.

# Supplementary Figure S4. Time to onset is descriptive because date availability was selective

(A) Empirical cumulative TTO distributions among reports with complete, logically valid dates. (B) Characteristics of reports with and without usable dates. No censoring, incidence, or survival-risk interpretation was applied.
